# Follow-up ecological studies for cryptic species discoveries: Decrypting the leopard frogs of the eastern U.S.

**DOI:** 10.1371/journal.pone.0205805

**Published:** 2018-11-09

**Authors:** Matthew D. Schlesinger, Jeremy A. Feinberg, Nathan H. Nazdrowicz, J.D. Kleopfer, Jeffrey C. Beane, John F. Bunnell, Joanna Burger, Edward Corey, Kathy Gipe, Jesse W. Jaycox, Erik Kiviat, Jacob Kubel, Dennis P. Quinn, Christopher Raithel, Peter A. Scott, Sarah M. Wenner, Erin L. White, Brian Zarate, H. Bradley Shaffer

**Affiliations:** 1 New York Natural Heritage Program, State University of New York College of Environmental Science and Forestry, Albany, New York, United States of America; 2 Department of Ecology, Evolution, and Natural Resources, Rutgers University, New Brunswick, New Jersey, United States of America; 3 Smithsonian Institution, National Museum of National History and Conservation Biology Institute, Washington, DC, United States of America; 4 Department of Entomology & Wildlife Ecology, University of Delaware, Newark, Delaware, United States of America; 5 Virginia Department of Game and Inland Fisheries, Charles City, Virginia, United States of America; 6 North Carolina Museum of Natural Sciences, Raleigh, North Carolina, United States of America; 7 New Jersey Pinelands Commission, New Lisbon, New Jersey, United States of America; 8 Division of Life Sciences, Rutgers University, Piscataway, New Jersey, United States of America; 9 North Carolina Division of Parks and Recreation, Raleigh, North Carolina, United States of America; 10 Natural Diversity Section, Pennsylvania Fish and Boat Commission, Bellefonte, Pennsylvania, United States of America; 11 New York State Office of Parks, Recreation, and Historic Preservation, Staatsburg, New York, United States of America; 12 Hudsonia, Ltd., Annandale, New York, United States of America; 13 Natural Heritage & Endangered Species Program, Massachusetts Division of Fisheries & Wildlife, Westborough, Massachusetts, United States of America; 14 CTHerpConsultant, LLC, Southington, Connecticut, United States of America; 15 Division of Fish and Wildlife, Rhode Island Department of Environmental Management, Providence, Rhode Island, United States of America; 16 Department of Ecology and Evolutionary Biology, University of California, Los Angeles, Los Angeles, California, United States of America; 17 Endangered and Nongame Species Program, New Jersey Division of Fish and Wildlife, Clinton, New Jersey, United States of America; 18 UCLA La Kretz Center for California Conservation Science, Institute of the Environment and Sustainability University of California, Los Angeles, Los Angeles, California, United States of America; University of South Dakota, UNITED STATES

## Abstract

Cryptic species are a challenge for systematics, but their elucidation also may leave critical information gaps about the distribution, conservation status, and behavior of affected species. We use the leopard frogs of the eastern U.S. as a case study of this issue. We refined the known range of the recently described *Rana kauffeldi*, the Atlantic Coast Leopard Frog, relative to the region’s two other leopard frog species, conducted assessments of conservation status, and improved methods for separating the three species using morphological field characters. We conducted over 2,000 call and visual surveys and took photographs of and tissue samples from hundreds of frogs. Genetic analysis supported a three-species taxonomy and provided determinations for 220 individual photographed frogs. *Rana kauffeldi* was confirmed in eight U.S. states, from North Carolina to southern Connecticut, hewing closely to the Atlantic Coastal Plain. It can be reliably differentiated in life from *R*. *pipiens*, and from *R*. *sphenocephala* 90% of the time, based on such characters as the femoral reticulum patterning, dorsal spot size and number, and presence of a snout spot. However, the only diagnostic character separating *R*. *kauffeldi* from *R*. *sphenocephala* remains the breeding call described in 2014. Based on our field study, museum specimens, and prior survey data, we suggest that *R*. *kauffeldi* has declined substantially in the northern part of its range, but is more secure in the core of its range. We also report, for the first time, apparent extirpations of *R*. *pipiens* from the southeastern portion of its range, previously overlooked because of confusion with *R*. *kauffeldi*. We conclude with a generalized ecological research agenda for cryptic species. For *R*. *kauffeldi*, needs include descriptions of earlier life stages, studies of niche partitioning with sympatric congeners and the potential for hybridization, and identification of conservation actions to prevent further declines.

## Introduction

Conservation biologists agree that a clear understanding of a region’s species is essential for biodiversity conservation [1–3]. Knowledge of the status, distribution, and delimitation of individual species within a region is vital for management, as species have distinct habitat needs, ecologies, and behaviors that often require specific policies and management considerations [4–7]. Biological inventories combined with detailed natural history observation are important tools for this, not just to enumerate a region’s species, but also—in some cases—for revealing evidence of new species. Among new species discoveries, cryptic species, defined as species incorrectly grouped under a single taxonomic name, are particularly relevant in areas that are seemingly well understood [8]. In most cases, cryptic species represent two or more species that are morphologically similar and easily confused with one another, but are phylogenetically distinct. For frogs as well as some other vocalizing taxa (e.g., insects, birds), otherwise cryptic species can often be differentiated by their audial signature, and bioacoustic analysis is an important tool for recognizing new or overlooked species [9]. Investigations have uncovered cryptic species in a variety of settings, including surprising locations and among presumably well-known taxa [8,10–12].

The resolution of cryptic species can have ramifications that extend far beyond systematics. When the dust settles and the taxonomy is sorted out, many fundamental questions remain about the ecology, evolution, and conservation of most cryptic species complexes, including 1) What is the distribution of each species? 2) How reliably can the species be distinguished from one another in the field? 3) What bearing does the discovery of one have on our knowledge of the other(s)? 4) Can we reconstruct the historical distributions of each species from museum specimens alone? 5) What is the conservation status of each species? 6) Do cryptic species occur allopatrically or sympatrically with their congener(s)? If they are sympatric, is there niche partitioning, competition, or hybridization associated with range overlap? 7) Do the species have different ecological roles, including their potential role in ecosystem services? 8) For closely related species, what evolutionary mechanism led to speciation? and 9) Do the answers to these questions alter our interpretation of the scientific literature on the revised species complex? Our view is that the systematic clarification of a cryptic species complex is only a first step that must be followed by further investigation. Otherwise, species-specific management and conservation actions will almost certainly be misapplied, potentially to the risk of one or more species.

Several recent studies have followed cryptic species discoveries to attempt to answer some of the questions above, with informational needs varying by the nature of the different species complexes being explored. The recent split of trilling chorus frogs (*Pseudacris* spp.) and identification of a new species [13,14], for example, prompted local status assessments based on the newly identified species limits and demonstrated sharp declines in one of the contained species [15,16]. Confirmation of cryptic diversity in cavefish [17] was followed by conservation status assessments that showed many lineages to be of conservation concern [18]. And Trillo et al. [19] followed the elucidation of cryptic diversity in Amazonian frogs [20] with a study of behavioral isolating mechanisms.

In this paper, we use the leopard frog species complex, with particular focus on the coastal northeastern United States, to illustrate the need for ecological research following a cryptic species discovery. In doing so, we present the first range-wide study of the newly described Atlantic Coast Leopard Frog (*Rana* [*Lithobates*] *kauffeldi*) along with new information on its two well-known regional congeners, the northern (*R*. *pipiens*) and southern (*R*. *sphenocephala*) leopard frogs. In the spirit of taxonomic stability, we retain the genus *Rana* for these frogs, following other recent publications on these species [21,22] that have rejected *Lithobates* based on currently available data.

### Focal taxa

Although the existence of three distinct northeastern leopard frogs has been well demonstrated through recent molecular and bioacoustical research [21,23], the natural history of these species has remained uncertain since the discovery of *R*. *kauffeldi*. This recent uncertainty is pre-dated by a long history of earlier confusion: despite the fact that some herpetologists had suggested that there might be undocumented leopard frog species in the northeastern U.S. [24–26], all of the region’s leopard frogs were considered *R*. *pipiens* for a substantial part of the previous century. Eventually that single-species framework gave way to a two-species taxonomy in the eastern US (adding *R*. *utricularia*, later renamed *R*. *sphenocephala*) following Pace [27]. Decades later, Newman et al. [23] documented a distinct genetic lineage in leopard frogs from northern New Jersey, southern New York, and central Connecticut, and Feinberg et al. [21] formally described this new species as *R*. *kauffeldi*, reporting a broader range (Connecticut to North Carolina) documenting for the first time differences in the primary mating call, morphology, and color pattern compared to either *R*. *pipiens*, mainly to the north, and *R*. *sphenocephala*, whose more southerly range appeared to overlap partially with that of *R*. *kauffeldi*.

The preliminary range map in Feinberg et al. [21] was based on several lines of evidence: genetics from the New York City metropolitan area, museum specimens whose locality and physical appearance could be reliably associated with each species, and bioacoustical sampling from as far south as North Carolina. Those authors used data from three sites with co-located bioacoustics and genetic information to support their conclusion that the distinct mating call they documented was from a genetically distinct species. In addition, Feinberg et al. [21] built on previous observations of leopard frog patterning and morphology [24,25,27,28] to propose a set of characteristics for reliable distinction among the three species. Feinberg et al. [21] concluded that additional research was needed to ensure that the patterns reported based on their few samples were reliable range-wide, and so could be used to resolve areas of distributional uncertainty across the entire putative range of *R*. *kauffeldi* and to confirm characters that could be used to reliably distinguish animals in the field without genetics or bioacoustics.

In this paper, we provide a particularly complete example of the follow-up research that is necessary when the taxonomy is sorted out for a cryptic species complex, using the leopard frogs of the eastern U.S. as a case study. We refined the range of *R*. *kauffeldi* relative to the two other recognized leopard frog species in the region so that managers and researchers in the ten states represented by our authorship team were better informed about which species were present in their state, and mapped new, ecologically informed ranges for all three taxa. Updated knowledge of the three species’ distributions then informed our updated assessments of conservation status, which were especially important in areas where leopard frogs (*sensu lato*) are already of concern. Finally, we used confirmatory genetic data to improve methods for separating the three species using acoustical and morphological field characters that should facilitate future inventory, monitoring, status assessments, and conservation actions.

## Methods

Field work conducted in 2014 and 2015 consisted of call and visual surveys to identify populations of each species, followed by sampling frog tissue for genetic analysis. We supplemented this work with examination of museum specimens and compilations of older survey data and a few more recent observations. Our study area was the entire potential range of *R*. *kauffeldi* as mapped by Feinberg et al. [21], encompassing the northeastern portion of the Atlantic Coastal Plain plus southern New England. Sampling occurred in the following U.S. states: Connecticut (CT), Delaware (DE), Maryland (DE), Massachusetts (MA), New Jersey (NJ), New York (NY), North Carolina (NC), Pennsylvania (PA), Rhode Island (RI), and Virginia (VA).

Permits for frog handling and tissue collection were issued by the following agencies: New York State Department of Environmental Conservation, Pennsylvania Fish and Boat Commission, Massachusetts Division of Fisheries and Wildlife, North Carolina Wildlife Resources Commission, Connecticut Department of Energy and Environmental Protection, Delaware Division of Fish and Wildlife, and Maryland Department of Natural Resources. In the other states special collection permission was unnecessary per state regulations or the authority granted state biologists. In states where leopard frogs had legal protection, our permits specifically referenced our permission to work on protected species.

Most calling surveys were conducted from roads or other public access points, and tissue collections were made from roads and from occupied habitats. Special permissions needed for site access were granted by the following agencies and organizations: U.S. Fish and Wildlife Service, The Nature Conservancy, New York State Office of Parks, Resources, and Historic Preservation, Orange County Land Trust (NY), Connecticut Department of Energy & Environmental Protection, Delaware Department of Conservation and Natural Resources, Bucks County (PA) Department of Parks and Recreation, New Jersey Division of Parks and Forestry, New Jersey Division of Fish and Wildlife, New Jersey Audubon Society, and New Jersey Conservation Foundation. For locations on private land, permission was granted by individual landowners prior to sampling.

### Call and visual surveys

To locate populations of leopard frogs and identify sites for subsequent sampling, we conducted call surveys in a variety of wetland habitats throughout the study area. Dates of surveys ranged from late February to late March in southern latitudes and from late April to early June in northern latitudes in 2014 and 2015. Because many survey partners had existing frog monitoring programs, and volunteers comprised a portion of the workforce, we allowed for considerable flexibility in survey methodology. At a minimum, observers recorded the GPS coordinates of sampling locations, time spent listening, and species detected. Sampling locations were selected by observers based on habitat suitability, access, and safety considerations. Large wetlands could have multiple sampling locations. Observers were asked to spend a minimum of 3 minutes (though some spent fewer) at each sampling location and to record the survey duration. If survey duration was not recorded, we assumed that it was 3 minutes. Surveys began no earlier than one half-hour after sunset and ended by 1:00 a.m. Surveys were not conducted when temperatures were below 4 degrees Celsius or in heavy rain or high wind. Observers made audio recordings of calls of suspected leopard frogs and the acoustically similar (to *R*. *kauffeldi*) Wood Frog (*R*. *sylvatica*) for subsequent confirmation from members of the team most familiar with the species.

From spring through fall in 2014 and 2015, we visited sites where we had confirmed the presence of leopard frogs bioacoustically to capture frogs for photographic and genetic analyses. We also visited sites of unknown occupancy and suspected historical occupancy for visual surveys to determine presence. If we encountered leopard frogs, we captured and photographed them following standardized digital imaging protocols (below), clipped the last digit of one toe as a tissue samples, stored the sample in 95% ethanol and shipped them to the University of California, Los Angeles (UCLA) for genetic analysis. We followed the State University of New York College of Environmental Science and Forestry’s Institutional Animal Care and Use Committee protocol #140102, developed specifically for this project, for the temporary care and handling of captured frogs.

### Photography

**Fig 1:**
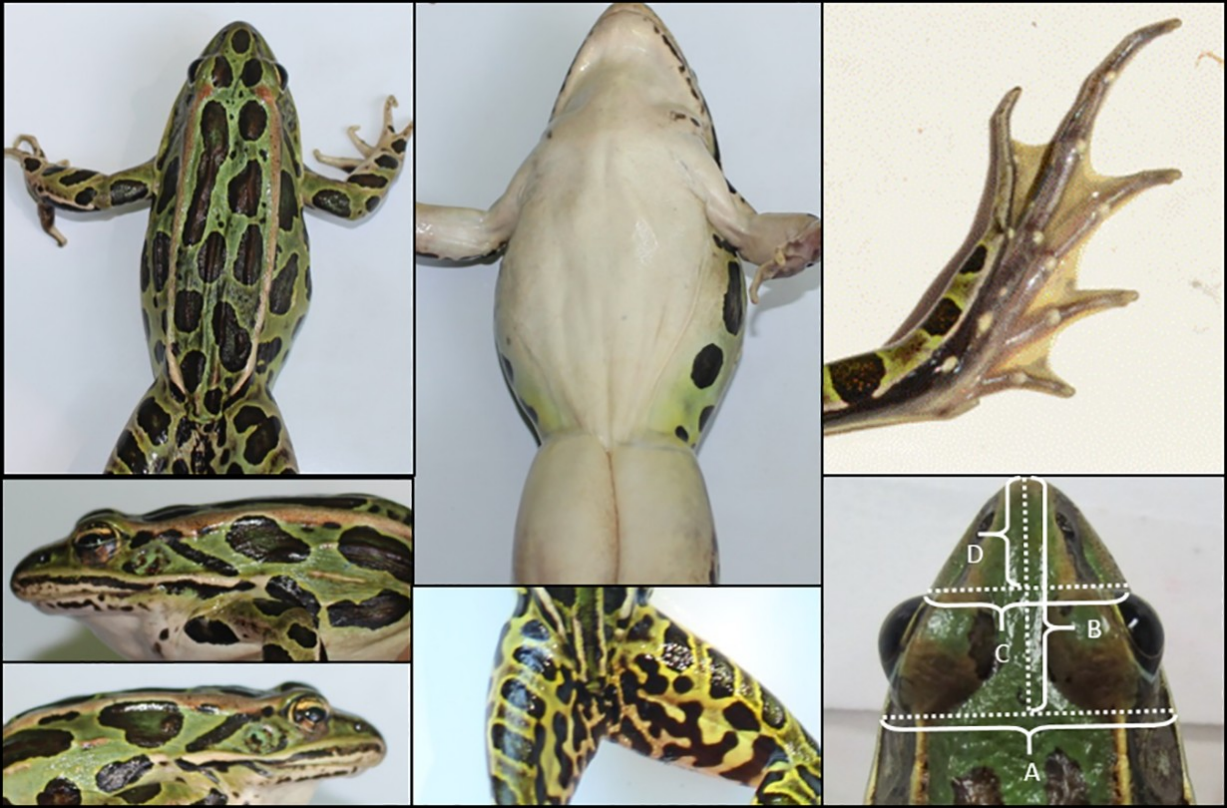
Example of photographs of a captured leopard frog taken from prescribed angles (clockwise from lower left): right side, left side, dorsal, ventral, femoral reticulum, hind foot; and photo measurements (lower right) of head width (A), head length (B), snout width (C), and snout length (D).

To aid in the identification of potentially reliable field characters, we photographed captured frogs from several specific angles (Fig 1) to clearly show the dorsal and ventral surfaces, snout profile, femoral reticulum, tympanum (right and left), and hind foot toe webbing, and matched each image with its corresponding tissue sample. This allowed us to explore many possible diagnostic features, including some of those referenced by Feinberg et al. [21] as well as earlier studies by Porter [29] and Moore [28].

We used our photographic data set to test the validity of these characters across the suspected range of *R*. *kauffeldi*. A single observer (ELW) with no explicit knowledge of character states suspected to differentiate these species evaluated photos of each genotyped frog to assess 1) number of dorsal spots from snout to vent occurring between the dorsolateral folds (conjoined spots were counted as two spots); 2) snout spot (large, small, or absent); 3) snout shape (blunt, pointed, or intermediate); 4) reticulum coloration (predominantly dark, predominantly light, or intermediate); 5) reticulum pattern (mostly large, connected blotches; or mostly small, unconnected dots); 6) left and right tympanum spot pattern (sharp dot, sharp blotch, or present but indistinct); 7) left and right tympanum spot color (white/cream, green, or brown/bronze); and 8) webbing on the first toe of the left and right hind food (curves all the way to the tip or stops about halfway to the tip). For all characters evaluated qualitatively, the observer had exemplar photographs to guide in interpretation (S1 Appendix).

We also used our dorsal photographs of genetically confirmed *R*. *kauffeldi* and *R*. *sphenocephala* to quantify snout shape and reticulum coloration, since these characters had been suggested previously to help distinguish these two species [21,29]. For snout shape, we imported each photo into ArcGIS 10.3 [30] and drew lines in four locations (Fig 1): A) along the posterior edge of the eyes, perpendicular to the spine of the frog, connecting the visible edges of the head (“head width”); B) perpendicular from the tip of the snout to line (A) (“head length”); C) along the anterior edge of the eyes but otherwise as in (A) (“snout width”); D) from the tip of the snout to line (C) (“snout length”). We recorded the length of each line in arbitrary map units, and calculated three ratios: head length to head width (B/A), snout length to snout width (D/C), and snout width to head length (C/B). We characterized a subset of reticula using image processing software (S2 Appendix).

### Genetic analysis

Genomic DNA extraction and PCR amplification were performed at UCLA following the methods in Newman et al. [23]. We amplified the nuclear genes neurotrophin-3 (NTF3, 599 bp), tyrosinase (Tyr 557–585 bp), Rag-1 (647–683 bp), seven-in-absentia (SIA, 362–393 bp), and chemokine receptor 4 (CXCR4, 550 bp). PCR products were bidirectionally sequenced at Beckman Coulter Genomics (Danvers, MA, USA). Contigs were assembled and trimmed in Geneious version 6.1.6 [31]. Consensus sequences for each locus were aligned with sequences from Newman et al. [23] using ClustalW in Geneious and manually adjusted.

To determine how all of the samples grouped into genetic population clusters, we used Structure version 2.3.2 [32,33] with an allelic data set (12–15% missing data) derived from our sequence data. We inferred haplotypes for each locus with a Bayesian algorithm using Phase version 2.1.1 [34,35]. Each allele was represented by a single haplotype. Phase input files were formatted from NEXUS files using a Perl script (RC Thomson, unpublished).

Following the same parameters as Newman et al. [23], we used Structure to determine the number of genetically distinct clusters (K) in our complete data set. We used the admixture model [32] and assumed correlation of allele frequencies among clusters and no other *a priori* population information. We ran 20 iterations of K values from 1 to 10, each with 100,000 generations plus a burn-in of 100,000 generations. We chose the appropriate K value by obtaining likelihood scores using the Evanno method [36]. We then assigned species identifications based on their genetic makeup: frogs were designated as “pure” if they had a 90% or greater match with a single species, while those with a species match between 10% and 90% were designated as “admixed.” We confirmed the robustness of genetic assignment of individuals to PHASE parameter choice and that these results were further validated with likelihood-based phylogenetic methods (S3 Appendix).

### Observation data and museum records

To supplement our field work and inform our reconstructions of the historical and current ranges of each species, we gathered observational and survey data from a variety of sources, including those outside the project team in cases where call recordings were available to confirm species identification. To determine the historical ranges of the three leopard frogs, we examined 2,522 museum specimens from the following institutions: American Museum of Natural History, Carnegie-Mellon Museum of Natural History, Cornell University Museum of Vertebrates, Harvard Museum of Comparative Zoology, National Museum of Canada, North Carolina Museum of Natural Sciences, Southern Connecticut State University, Smithsonian National Museum of Natural History, Texas Cooperative Museum, University of Connecticut, and Yale Peabody Museum of Natural History. Based on identification characters from Feinberg et al. [21] and our own findings, we determined probable species identifications that we used to inform maps of historical ranges. In a small percentage of cases (1–2% of specimens examined) where color patterns had faded or the typical characters used to determine species were unclear, we either made our best informed identification or did not assign a species; these few specimens did not substantially affect our historical distribution assessments. Most or all of these historical specimens had been preserved in formalin, making molecular examination challenging [37].

### Data analysis

#### Call surveys and genetics

To test whether the unique call described in Feinberg et al. [21] was associated with frogs genetically identified as *R*. *kauffeldi*, we examined the results of call surveys in the vicinity of locations of genetic samples for the three species. It was not feasible to match calls to genetics at the individual level because of difficulties with collecting tissue from specific, elusive callers in often impenetrable coastal marshes. Instead, we matched calls to genetics at the population level by comparing the genetic identity of sampled frogs (“pure” individuals only) to the results of nearby call surveys (within 100 m and 300 m of each genetically sampled frog).

#### Morphological and color characters

We report simple summary statistics and frequencies of different character states for each species. To test for differences among species for particularly important or challenging characters, we used one-way ANOVAs in R version 3.0.2 [38]. In case no single character was diagnostic in distinguishing among species, we used multivariate methods to explore the value of combinations of characters in identifying the species. We used the randomForest package [39] in R version 3.0.2 [38] to run a random forests classification analysis [40] on the 16 characters obtained from photos with the genotyped species identification as the dependent variable. Only “pure” individuals were included. Missing values, resulting from photos of certain angles not being submitted or inconclusive images, were treated by running two separate analyses: one in which we imputed them using the function rf.impute and another in which we omitted cases with missing values for some characters. For each analysis, we built 1,000 trees and determined variable importance as the mean decrease in model accuracy without each variable following Strobl et al. [41].

#### Species distribution mapping and modeling

Our final maps of species occurrence are based upon genotyped frogs from this study and Newman et al. [23], call surveys from this study and Feinberg et al. [21], incidental documentation of calling frogs by the authors, and other recorded calls or confirmed visual identifications (mainly from 2005–2013, but as far back as the 1990s and some from 2016 and 2017). We developed current range maps for each of the three northeastern leopard frog species using these presence points in combination with previous range maps [21,42] and watershed boundaries. We then used these maps to develop historical range maps (for *R*. *kauffeldi* and *R*. *pipiens* only) by incorporating museum specimens for which we felt confident in our species determination.

We built a “presence-only” species distribution model for *R*. *kauffeldi* following methods in Howard and Schlesinger [43]. In brief, we attributed 169 presence points and 10,000 background points with 81 environmental layers representing climate, geology, topography, and land cover using 30-m grid cells (T. Howard, NY Natural Heritage Program, unpublished). Background points were restricted to a 171,704-km^2^ modeling area that encompassed the area of known presence, matching 8-digit Hydrological Unit Code boundaries [44]. We used the randomForest package [39] in R version 3.0.2 [38] to run a classification analysis [40] to distinguish areas of predicted presence of suitable habitat from areas of predicted lack of suitable habitat. We used the “out-of-bag” estimate of the error rate [40] and the confusion matrix as measures of the model’s accuracy, determined variable importance following Strobl et al. [41], and used the results of the analysis to predict the probability of suitable habitat for the modeling area.

### Determining conservation status

To help prioritize conservation and management actions, we suggested conservation status ranks for *R*. *kauffeldi* for its entire range and for each state in which it was documented. We used the NatureServe and Natural Heritage Program methodology [45,46], to determine conservation status by evaluating a suite of factors representing rarity, threats, and trends to arrive at a G-rank (“global” rank, for the entire range; Table 1) or S-rank (for a state or other subnational jurisdiction). We focused on three rarity factors that we felt our data could best inform—range extent (area encompassed by the outer boundary of presence points), area of occupancy (number of 4-km^2^ grid cells occupied), and number of occurrences (estimated by counting a detection as a separate occurrence if separated by 5 km of suitable habitat or 1 km of unsuitable habitat; [47]). For each of these categories, the methodology uses wide value ranges and provides the opportunity to select multiple scores to encapsulate uncertainty.

**Table 1:** Conservation status ranks used in the NatureServe methodology [45,46]. From http://www.natureserve.org/conservation-tools/conservation-status-assessment. At the subnational level, S-ranks are used.

Global Rank	Definition
G1	Critically Imperiled—At very high risk of extinction due to extreme rarity (often 5 or fewer populations), very steep declines, or other factors.
G2	Imperiled—At high risk of extinction or elimination due to very restricted range, very few populations, steep declines, or other factors.
G3	Vulnerable—At moderate risk of extinction or elimination due to a restricted range, relatively few populations, recent and widespread declines, or other factors.
G4	Apparently Secure—Uncommon but not rare; some cause for long-term concern due to declines or other factors.
G5	Secure—Common; widespread and abundant.
GX	Presumed Extinct—Species not located despite intensive searches and virtually no likelihood of rediscovery. Ecological community or system eliminated throughout its range, with no restoration potential.
GH	Possibly Extinct (species)—Known from only historical occurrences but still some hope of rediscovery. There is evidence that the species may be extinct or the ecosystem may be eliminated throughout its range, but not enough to state this with certainty.

Our data were less well suited to addressing threat and trend factors, largely due to the lack of species-specific information on threats. Thus, we used the optional factor intrinsic vulnerability, a surrogate for threats representing susceptibility to impacts from human activities based on life history. Amphibians are highly sensitive to disease [48] and aquatic pollutants [49,50], and *R*. *kauffeldi*’s proximity to the coast makes it vulnerable to habitat degradation and loss from coastal storms and rising sea levels [21,51]. *Rana kauffeldi* and *R*. *sphenocephala* were both found to be susceptible to several amphibian diseases (including chytridiomycosis, ranavirus, and a perkinsus-like organism) when reared in outdoor pens on Long Island, in wetlands where leopard frog complex members are now extinct (JAF, unpublished data). On the other hand, *R*. *kauffeldi* is known from wetlands near heavy industry [21], suggesting it may be relatively insensitive to environmental toxins if suitable freshwater habitat exists. Some of these industrial wetlands in southern NY and northern NJ are threatened by development. We characterized intrinsic vulnerability as the combination rating “High or Moderate” for all jurisdictions to reflect the balance between these susceptibilities and apparent tolerances.

Information on trends was mostly lacking given the recent discovery of this species and the difficulty of identifying older museum specimens with certainty. We calculated a G-rank for the overall range and S-ranks for each state in two ways: using NatureServe’s element rank calculator [52] and based on our interpretation of the data.

## Results

### Call surveys

**Fig 2:**
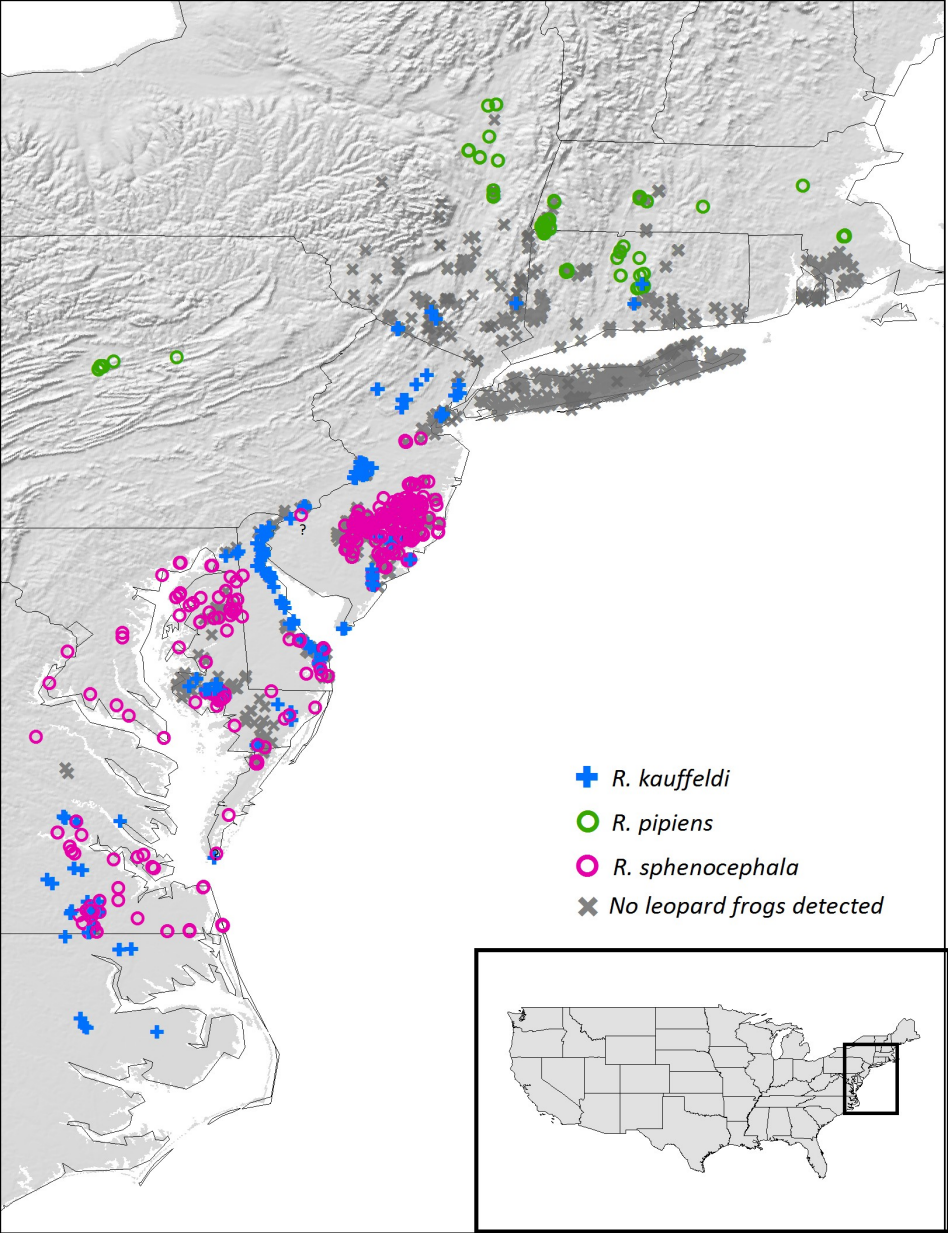
Leopard frog presence and non-detections from call surveys and genetic analysis in the northeastern U.S. Question marks are directly below presence points where identifications from genetic analysis were questionable. Shaded relief map from https://nationalmap.gov/.

We conducted call surveys at 1,004 point locations throughout the northeastern U.S. (Fig 2). Most points were surveyed once, with some surveyed multiple times, for a total of 2,159 surveys. Survey durations ranged from 1 to 110 minutes (mean = 5.76 minutes) and totaled approximately 207 hours. Surveys were conducted between 21 February 2014 and 15 June 2015. Additional call survey data dating back to 2006 were included as available.

Calls from *R*. *kauffeldi* were documented as early as 10 March 2014 in MD and 14 March 2015 in DE. Outside the formal window of our field work, calls were documented on 3 February 2016 in NC, 21 February 2017 in VA, and 8 March 2016 in NY. The last call dates for *R*. *kauffeldi* in the two project years were 23 April 2014 in PA and 15 April 2015 in NJ. Calls have been heard sporadically in late April and May in NY (JAF, unpublished data), and 12 June 2006 (with the identification confirmed later) in NJ’s Meadowlands [53].

### Genetics

We collected tissue samples from 254 individual frogs for genetic analysis from throughout the putative range of *R*. *kauffeldi* and beyond to include samples of all three species across the region. Of these samples, 251 were successfully extracted, amplified, and sequenced. Three samples had DNA concentrations that were too low for successful PCR amplification and were removed from the analysis. Aligned sequence lengths for nuclear loci were similar to those for a broader set of leopard frog samples for the same loci [23]: 527 bp (CXCR4), 530 bp (NTF3), 648 bp (Rag-1), 388 bp (SIA), and 549 bp (TYR). There were between 31 and 49 variable sites per locus. Individuals that received greater than three null haplotypes were removed from the analysis. In total, 243 of the original 251samples plus 50 individuals determined to be pure *R*. *kauffeldi*, *R*. *pipiens*, or *R*. *sphenocephala* by Newman et al. [23] went into the final analysis, for a total of 293 frogs. The results of Structure analyses from both the 80% and 90% probability Phase data sets were quantitatively similar (S3 Appendix); given that the 80% Phase probability set was slightly larger, we use it in the remaining analyses.

Bayesian cluster analysis in Structure resolved three clusters (lnL = 4314.60, DK = 130.93). Individuals with a cluster probability greater than 90% were assigned to that cluster, and individuals with a cluster assignment between 10% and 90% were designated as admixed. We assigned species identification to each cluster using the individuals from Newman et al. [23] as reference samples. A total of 262 individuals fell into one of the three clusters unambiguously, including 111 *R*. *kauffeldi*, 79 *R*. *sphenocephala*, and 72 *R*. *pipiens* (Fig 2). The remaining 32 individuals were considered potential hybrids that we identified by the dominant species based on admixture proportions.

The concatenated maximum likelihood phylogeny identified three major, but poorly supported, clades representing *R*. *kauffeldi*, *R*. *pipiens*, and *R*. *sphenocephala*. There were also a few individuals that could not be attributed to any of these three clades and were generally identified as admixed in the Structure analysis.

We identified and omitted from further analysis four samples that appeared, upon genotyping, to have identification or locality errors. Two samples from one site in central CT appeared to have their labels switched: one was visually *R*. *pipiens* but was genotyped as *R*. *kauffeldi*, while the other was visually *R*. *kauffeldi* but was genotyped as *R*. *pipiens*. Both species are known to occur at this site based on other individuals, and together with another site in central CT are the only known sympatric sites for these two species. Two additional samples are identified with question marks in Fig 2. One frog from the southern tip of the Delmarva Peninsula (Northampton Co., VA) appeared visually to be *R*. *sphenocephala* but was genotyped as *R*. *kauffeldi*. Only calls of *R*. *sphenocephala* have been documented in the area and appropriate habitat for *R*. *kauffeldi* does not appear to exist at that locality. An additional frog in western NJ near the Delaware River (Gloucester Co.) was genotyped as *R*. *sphenocephala*, but no other *R*. *sphenocephala* are known from that mesic area of the state. No photographs were associated with this sample, but another frog from the site appears visually to be *R*. *kauffeldi*. Leopard frogs from along the Delaware River (Delaware Co., PA) show field characters of both species, so we cannot rule out either species just across the river in NJ. All the above cases of confusion may be highly admixed frogs, and further investigation of these potential hybrid zones is certainly needed.

### Species distributions

#### Rana kauffeldi

**Fig 3:**
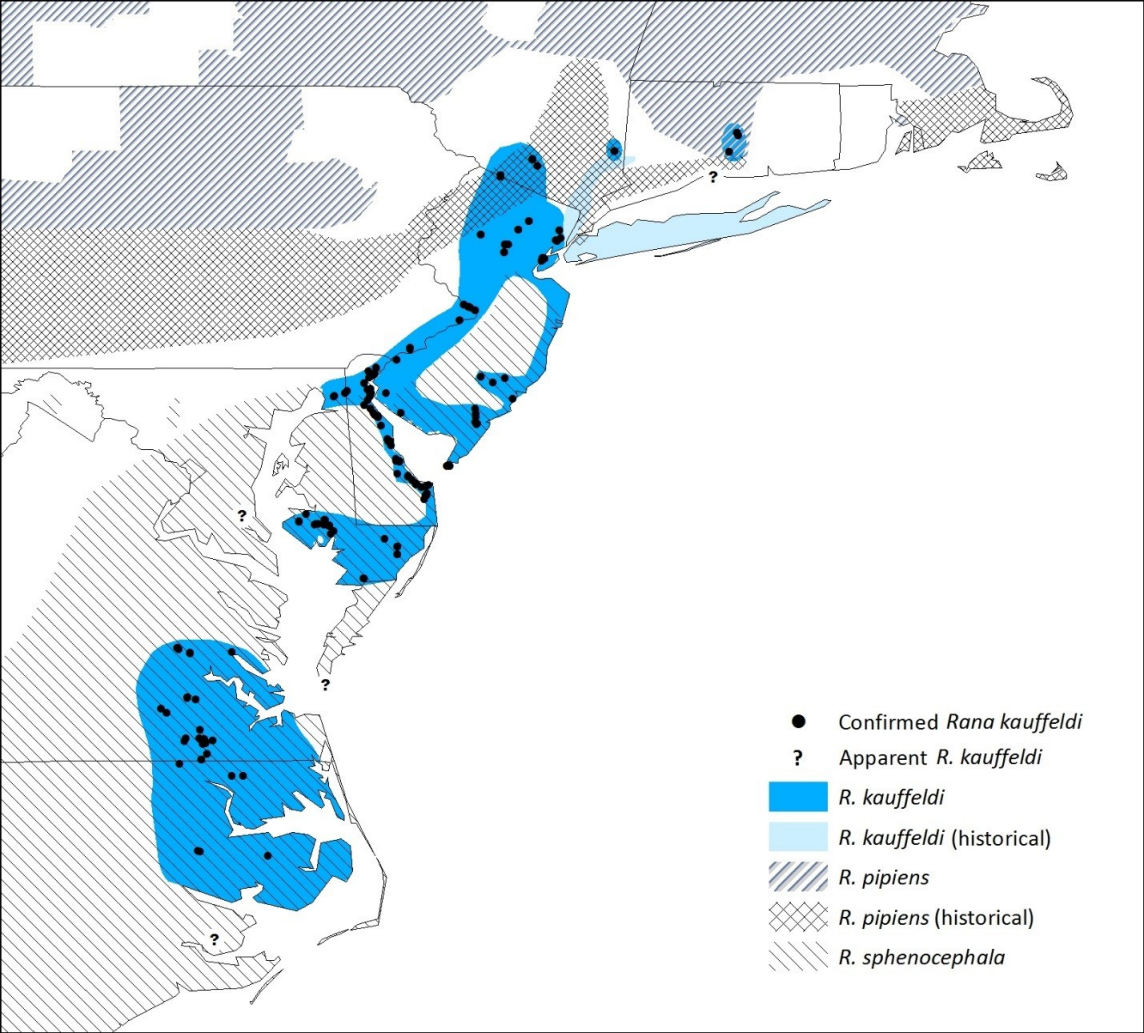
Range of *Rana kauffeldi*, with presence points confirmed by bioacoustics or genetics, compared to ranges of *R*. *pipiens* and *R*. *sphenocephala*. Historical ranges for *R*. *kauffeldi* and *R*. *pipiens* are based on examination of museum specimens and recent detections. Question marks denote locations where museum specimens appear to be *R*. *kauffeldi* but no bioacoustic or genetic evidence is available, or where genetic determinations were questionable.

We confirmed *Rana kauffeldi* in eight eastern U.S. states: CT, NY, NJ, PA, DE, MD, VA, and NC. We did not detect *R*. *kauffeldi* in MA or RI, where only *R*. *pipiens* was detected. The two locations farthest from one another in CT and NC are 746 km apart, close to the 780 km estimated by Feinberg et al. [21]. The range of *R*. *kauffeldi* that we estimate (Fig 3) covers just over 46,500 km^2^ from 208 m elevation in northern NJ to sea level in multiple locations. No *R*. *kauffeldi* were detected south of the 17°C isocline (i.e., annual average temperature greater than 17°C). Confirmed extremes of the range of *R*. *kauffeldi* were as follows (in WGS84): northernmost—Middlesex County, CT (lat 41.63, long -72.62); southernmost—Washington County, NC (lat 35.79, long -76.41); easternmost—Middlesex County, CT (lat 41.60, long -72.61); and westernmost—Sussex County, VA (lat 36.98, long -77.27). A frog collected in 1989 farther south in Hyde, NC (USNM 292558) appears to be *R*. *kauffeldi*, but we lack bioacoustic and genetic confirmation at that location.

The species does not occur far from coastally influenced habitats. The maximum distance the species was documented from the ocean, bays, and estuaries was 40 km near the border of NJ and NY. Eighty-nine percent of *R*. *kauffeldi* locations were within 20 km, 77% were within 10 km, and just under 50% were within 1 km of coastal waters.

In portions of its range, *R*. *kauffeldi* overlaps with its close congeners. *Rana pipiens* and *R*. *kauffeldi* were documented to be syntopic at the two aforementioned sites in CT, and the ranges of *R*. *sphenocephala* and *R*. *kauffeldi* overlap broadly from central NJ south to NC, including several instances of syntopy. In central NJ, DE, and MD, they appear to be less frequently sympatric than in VA and NC.

Our survey data also support the view promoted by Feinberg et al. [21] that *R*. *kauffeldi* has disappeared from a large part of its historical range in southern NY and CT (Fig 3), including much of the Hudson Valley and all of Long Island. We could not verify recent reports of leopard frogs from these areas despite considerable survey effort.

#### Rana pipiens

We report apparent extirpations of *R*. *pipiens* from the southern portion of its known range from PA east through northwestern NJ, southeastern NY, southern CT, southern RI, and coastal MA. We confirmed multiple museum specimens as *R*. *pipiens* from these regions, but our surveys and those of the Pennsylvania Amphibian and Reptile Survey [54], including many historical locations for *R*. *pipiens*, have not yielded leopard frogs of any species at those locations (Fig 2), with the exception of a single population near Providence, RI, discovered in 2017. The southernmost location at which we documented extant *R*. *pipiens* in the northeastern U.S. was in Middlesex County, CT (lat 41.49, long -72.69). Locations in lower latitudes have been reported by others in central PA [54] and farther west.

#### Rana sphenocephala

Prior range maps of *R*. *sphenocephala* (e.g., [42,55]) included southern NY and northern NJ. As suggested by Feinberg et al. [21], we confirmed that these areas are occupied, or were formerly occupied, by *R*. *kauffeldi* or *R*. *pipiens*, not *R*. *sphenocephala*. One possible exception is the xeric Pine Barrens of Long Island (NY), where leopard frogs were once common, but few museum specimens exist from this habitat to verify species composition. The northernmost extant locality for *R*. *sphenocephala* in our surveys (and apparently anywhere in its entire range) is in Middlesex County, NJ (lat 40.42, long -74.35).

### Match of calls to genetics

We found a near-perfect match of population-level calling with genetics of individual frogs. At 16 sites for *R*. *kauffeldi*, 18 sites for *R*. *sphenocephala*, and 3 sites for *R*. *pipiens*, genetically pure frogs of each species were confirmed where calling by that species was documented within 100 m; the only mismatch was one location (Sussex Co., DE) where *R*. *kauffeldi* was documented calling but the genetic identification of an individual from that location was *R*. *sphenocephala*. The same concordance between calls and genetics held for situations where calling frogs were documented within 300 m of genetic samples (23 sites for *R*. *kauffeldi*, 21 for *R*. *sphenocephala*, and 11 for *R*. *pipiens*); the only potential exceptions were three genetically *R*. *kauffeldi* sites that had both *R*. *kauffeldi* and *R*. *sphenocephala* calling, and the above-noted genetically *R*. *sphenocephala* site with *R*. *kauffeldi* calls.

### Morphological and color characters

We examined 912 photographs of 220 leopard frogs with genetic identities as follows: 80 pure *R*. *kauffeldi*, 16 admixed *R*. *kauffeldi*, 45 pure *R*. *pipiens*, 5 admixed *R*. *pipiens*, 64 pure *R*. *sphenocephala*, and 10 admixed *R*. *sphenocephala*. Not all 220 frogs had suitable photographs of all characters, so sample sizes for each character varied slightly. The comparisons below were based on examination of pure individuals only.

**Fig 4:**
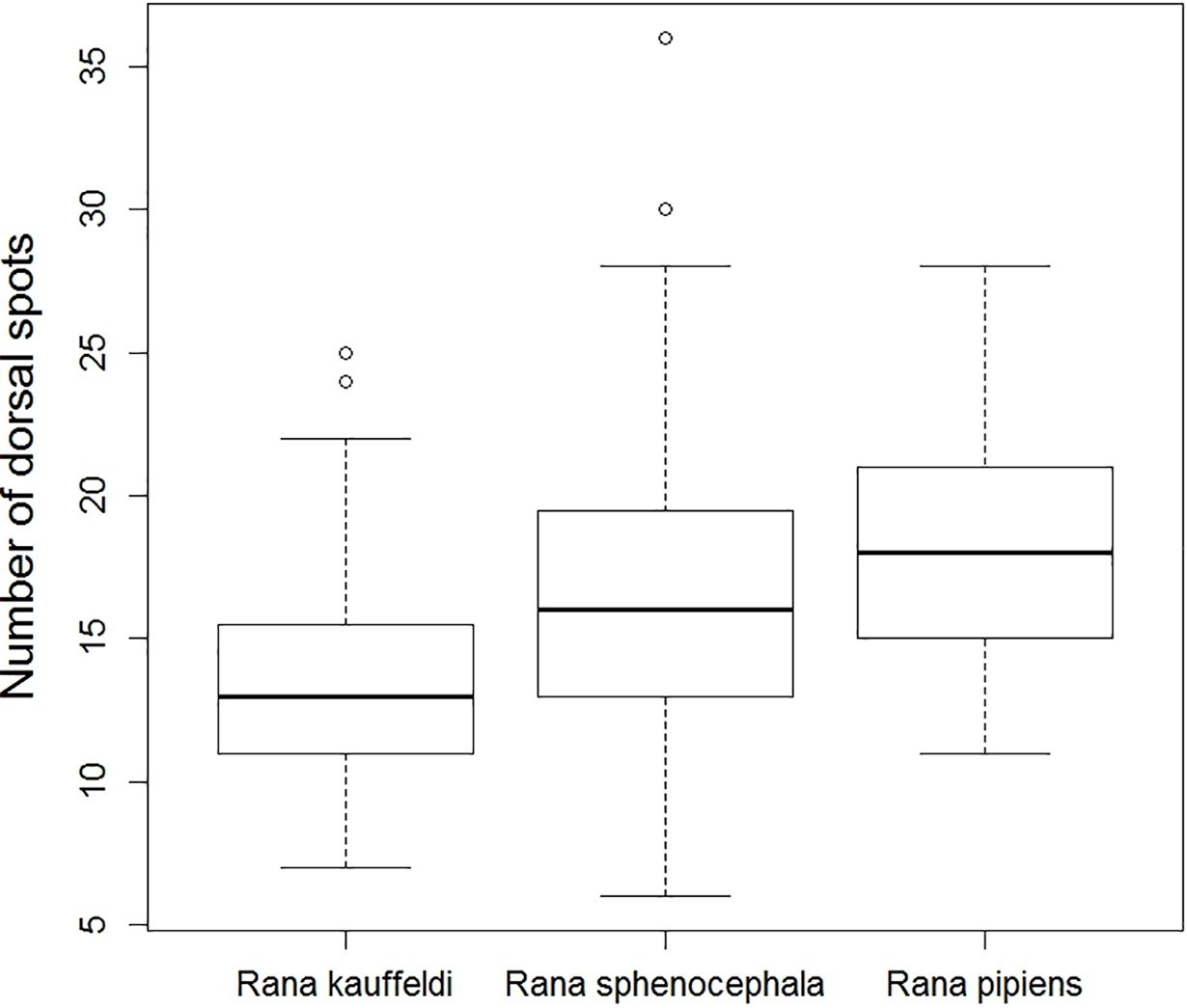
Boxplot of number of dorsal spots for three species of leopard frogs in the northeastern US. Open circles are statistical outliers.

*Rana kauffeldi* had fewer dorsal spots on average than both *R*. *pipiens* and *R*. *sphenocephala* in a one-way ANOVA (F_174,2_ = 15.08, *P* < 0.0001, with Tukey HSD post-hoc tests), although there was considerable overlap among species (S4 Appendix; Fig 4). *Rana sphenocephala* and *R*. *pipiens* had similar numbers of spots.

**Fig 5:**
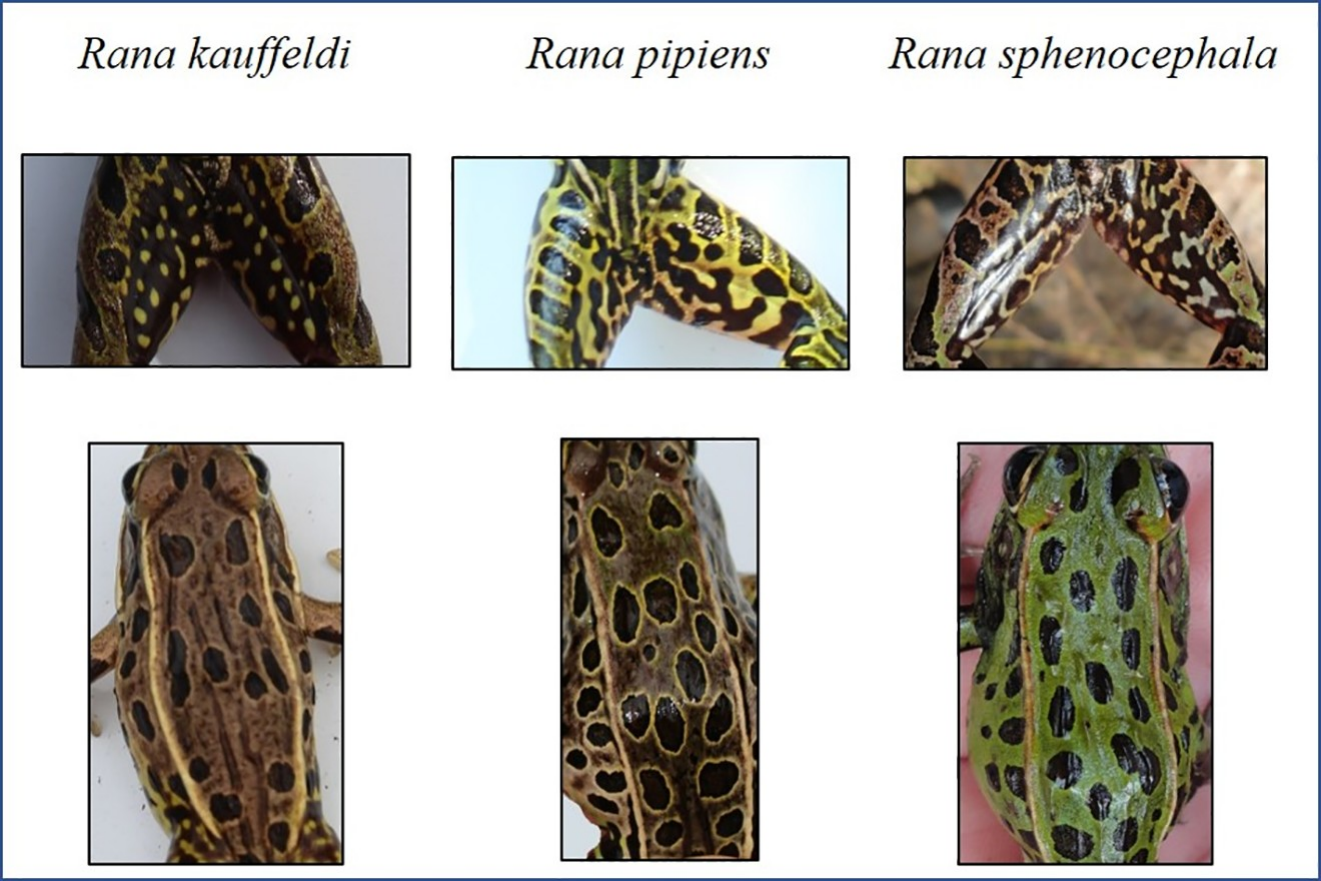
Typical patterns in femoral reticula (top row) and dorsal spotting (bottom row) in three species of leopard frogs in the northeastern U.S.

*Rana kauffeldi* was readily distinguished from *R*. *pipiens* by smaller body spots (85% of *R*. *kauffeldi* had spots smaller than the eye vs. 36% for *R*. *pipiens*), the frequent absence of a snout spot (85% absent vs. 29%), reticula characterized as predominantly dark (97% vs. 0%), and overall duller coloration (S4 Appendix; Fig 5).

*Rana kauffeldi* and *R*. *sphenocephala* were more challenging to differentiate, as no single character reliably distinguished the two species. Nearly all *R*. *kauffeldi* reticula appeared predominantly dark with small, unconnected dots of light pigment, while most *R*. *sphenocephala* reticula appeared predominantly light with large, connected blotches of dark pigment (S4 Appendix; Fig 5). The overall coloration of *R*. *kauffeldi* was typically duller than that of *R*. *sphenocephala*. *Rana kauffeldi* had sharp tympanum spots less frequently (58% of frogs) than *R*. *sphenocephala* (91%), and the snout of *R*. *kauffeldi* was more frequently characterized as “blunt” (62% of frogs) than that of *R*. *sphenocephala* (13%). The three head measurement ratios were not significantly different between the two species in a one-way ANOVA, although the ratio of snout width to head length was nearly so (F_121,1_ = 3.899, 0.05 < *P* < 0.10). The extent of toe webbing and the color of reticulum spots and blotches did not consistently differentiate the three species.

Characters used in combination provided accurate identification in most cases. Random forest analysis using 144 individuals with missing values imputed correctly classified *R*. *kauffeldi* and *R*. *sphenocephala* in over 90% of cases (out-of-bag estimate of error rate = 9.72%). The most important field characters in distinguishing between species were reticulum color, reticulum pattern, and snout shape. When the analysis was run with cases with missing values omitted (leaving n = 33 individuals), the error rate was similar (9.09%), although the order of important variables shifted somewhat, with ratio of snout width to head length, reticulum color, reticulum pattern, and ratio of head length to head width being most important.

Sample sizes of admixed individuals were insufficient for statistical analyses, but examination of the raw numbers (S4 Appendix) did not show the intermediacy of characteristics that would be expected to result from hybridization.

### Habitat association and distribution model

Our surveys found that the basic habitat description in Feinberg et al. [21] holds. South of the glacial maximum, *R*. *kauffeldi* is a habitat specialist restricted to large coastal and riparian wetlands. In the southern portion of its range, it occurs primarily in riparian cypress-gum swamps. Along the Delaware River and Bay, it occupies large freshwater impoundments, tidal guts, and tidal freshwater marshes often dominated by introduced Common Reed (*Phragmites australis*) marshes that may be subject to salinity intrusions. In the northern, recently glaciated portion of its range, *R*. *kauffeldi* typically occupies large freshwater wetlands with open canopies that otherwise are indistinguishable from similar wetlands where it was not detected. Where *R*. *sphenocephala* is sympatric with *R*. *kauffeldi*, *R*. *sphenocephala* is a generalist, being found in nearly any semi-permanent (isolated) or permanent wetlands, created or natural, including tire ruts, fish hatchery ponds, waterfowl impoundments, and cypress-gum swamps. It also appears to be less restricted to xeric habitats in the southern portion of its range than in more northerly locations (e.g., New Jersey).

**Fig 6:**
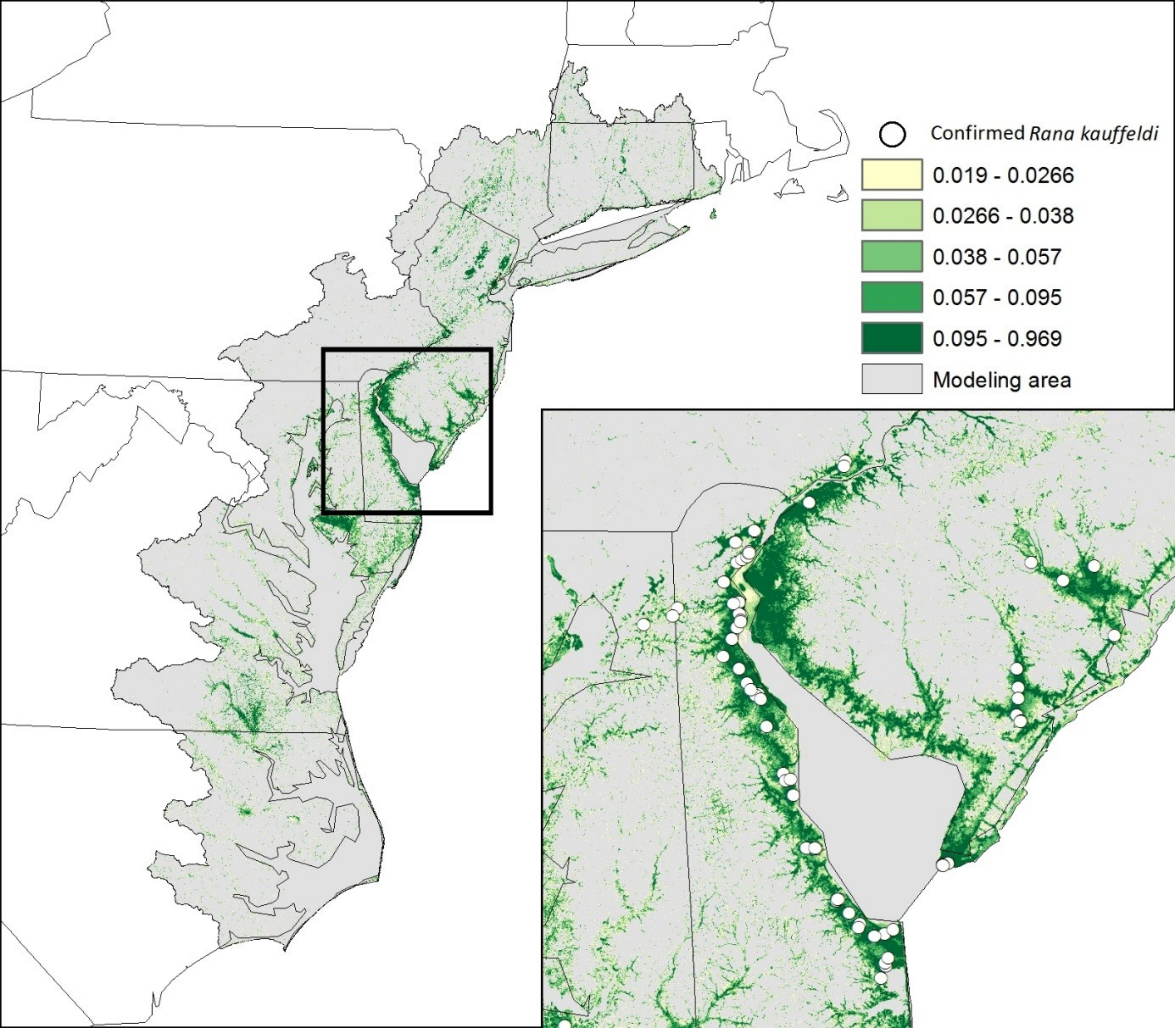
Distribution model for the full range of *R*. *kauffeldi* along the coastal northeastern U.S. Inset: Delaware Bay and surrounding states. Values reflecting habitat suitability were broken into 10 quantiles, with the top five displayed.

Our distribution model shows that suitable habitat for *R*. *kauffeldi* is concentrated along the coast and in riparian corridors (Fig 6). The model overall was very accurate, with an out-of-bag error estimate of 1.11%, although absences were predicted with greater accuracy (99%) than presences (34%). The factors most closely associated with the presence of *R*. *kauffeldi* were low elevation, high impervious surface and more wetlands in the surrounding landscape, and short distances to calcareous bedrock, saltwater or freshwater emergent wetlands, freshwater forested wetlands, and lakes and rivers.

### Conservation status of *Rana kauffeldi*

Because we recognized that a two-year survey, even when supplemented with pre-survey data, could not reveal all locations of *R*. *kauffeldi*, we embraced the uncertainty allowed by the NatureServe approach. For example, when our counts of number of grid cells or number of occurrences fell near the boundaries of ranking categories, we selected both categories. We identified a considerable decline in NY and CT based on our surveys and Feinberg et al. [21], but the evidence for statewide decline in other states within the range was weaker, so we did not use trends factors for those states. We estimated a slight range-wide decline based on our surveys (Table 2).

**Table 2:** Conservation status ranking of *Rana kauffeldi* using the NatureServe methodology [45,46]. Range extent is defined by the smallest polygon that encapsulates known occurrences. Area of occupancy is the area within the range in which the species actually occurs. Number of occurrences is intended to reflect number of populations, based on taxon-specific distances within which animals are assumed to be interacting. Long-term trend was estimated based on historical literature or museum specimens. Values given for these rank factors are the ranges encompassed by NatureServe’s categories. Calculated rank was generated by NatureServe’s element rank calculator [46], while Expert rank was assigned by biologists familiar with the species in that state.

Assessment area	Range Extent (km^2^)	Area of Occupancy (# 4-km^2^ cells)	# Occurrences	Long-term Trend^a^	Calculated Rank^b^	Expert Rank^b^
Rangewide	20,000–200,000	26–125	21–80	10–30% decline	G2G3	G3G4
CT	<100	3–5	1–5	NA	S1	S1
DE	1,000–5,000	26–125	6–80	NA	S2? ^c^	S4
MD	5,000–20,000	6–25	6–20	NA	S2	S3
NJ	5,000–20,000	26–125	6–80	NA	S2S3	S4
NC	1,000–20,000	6–25	1–20	NA	S1S2	S1S3
NY	250–5,000	6–25	1–20	50–90% decline	S1S2	S1S2
PA	100–250	2	1–5	NA	S1	S1
VA	1,000–20,000	6–125	6–80	NA	S1S3	S4

^a^ NA = not assessed

^b^ Status rank definitions are in Table 1

^c^ A? is used to denote uncertainty in the rank.

The ranks generated by the rank calculator tended toward greater concern than those based on our expert opinion (compare the last two columns in Table 2), but we report both to be transparent about our process. In the core of *R*. *kauffeldi*’s range (NJ, DE, VA, and perhaps MD), we believe it to be secure, with many apparently large populations in protected wetlands. At the northern edge of its range (including eastern PA, southern CT, southern NY, and extreme northeastern NJ), *R*. *kauffeldi* is exceedingly rare and appears to have declined substantially. In NY, for example, the species once occurred across 11 counties and likely more than 100 populations; today it is known from only three counties and fewer than 10 populations. Declines in NY’s leopard frogs have been suspected for decades [56]. While the species is common along the Delaware River, only a sliver of its range falls within PA, hence its suggested S1 status in that state. At the southern edge of our survey limits—and possibly the actual southern range limit for *R*. *kauffeldi*—in NC, it may be rare too, but this area needs additional field surveys to confirm the species’ status and actual southern limit. Throughout its range a rank of G3G4 (Vulnerable to Apparently Secure) seems appropriate.

## Discussion

### Decrypting *Rana kauffeldi*

Our multi-year, 10-state investigation demonstrated conclusively that *R*. *kauffeldi* is a habitat specialist with a small range centered in the most densely populated region of the United States, and one of the most heavily populated mega-regions on earth [57]. In several northern states, it is extremely rare, while in the southern portion of its range it is still broadly distributed and can be common. We have a much better idea of its distribution than we did just a few years ago, but some unexplained gaps remain. Fortunately, for those interested in surveying for this frog, methods for its identification in the field are also now better understood. The unique breeding call identified by Feinberg et al. [21] was reliably associated in our study with frogs genetically determined to be *R*. *kauffeldi*. Separation in the hand from *R*. *pipiens* based on morphology and color patterns is very reliable, while separation from *R*. *sphenocephala* is correct as much as 90% of the time.

#### Distribution and biogeography

**Fig 7:**
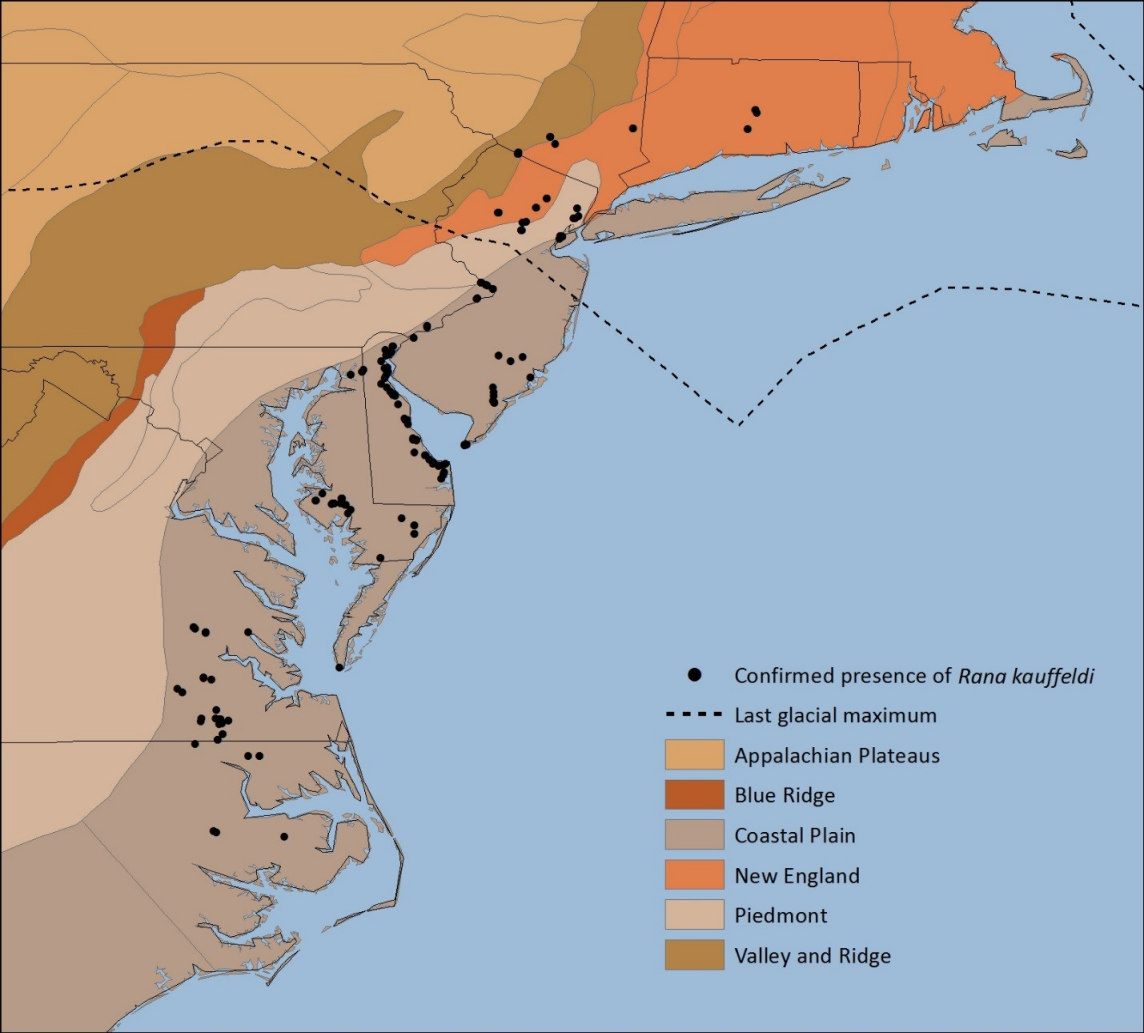
Physiographic regions of coastal northeastern U.S. ([58]; https://water.usgs.gov/lookup/getspatial?physio) with presence points for *Rana kauffeldi* confirmed by bioacoustics and/or genetics. Last glacial maximum is from Ray and Adams [63].

In the core of its mid-Atlantic range south of the Pleistocene glacial maximum, *R*. *kauffeldi* is a species exclusively of the Coastal Plain (Fig 7), and the degree to which its apparent western range margin matches that of the Coastal Plain is striking. The Coastal Plain physiographic region of the U.S. covers the Gulf and Atlantic coasts from southern Texas east to Florida and north to Long Island, NY [58]. The region is characterized by low elevation, minimal topography, and unconsolidated sediments [59] and has recently been recognized as a global biodiversity hotspot [60]. Within this physiographic region, several ecoregions—areas with similar geology, soils, climate, and vegetation [61]—have been identified, of which the Mid-Atlantic Coastal Plain (MACP), Chesapeake Bay Lowlands (CBY), and North Atlantic Coast (NAC) are occupied in part by *R*. *kauffeldi*. The MACP has been described as “a factory for the generation of new and novel species” because of its dynamism and juxtaposition of natural communities [62].

But since the last glacial maximum occurred only 20,000 years ago, *R*. *kauffeldi* has clearly moved out of its core region post-glacial retreat, (re)colonizing some previously glaciated regions along major river valleys to the north and east, and now occurs in the Piedmont, New England, and Valley and Ridge physiographic provinces. These ecoregions are characterized by greater topographic variation and a greater diversity of habitat types and soils than the core of the species range, perhaps reflecting the competitive or predator release that can characterize the opening of newly available habitat.

Apparent gaps in the range in areas with at least some suitable habitat—the northwestern shore of the Delmarva Peninsula and the western shore of the Chesapeake Bay from Baltimore, MD to mid-coastal VA—may be clarified with further sampling and continued examination of museum specimens and historical recordings. Examination of USNM museum specimens from the western shore of the Chesapeake Bay yielded little evidence of *R*. *kauffeldi* across that region apart from two specimens from Calvert County, MD that more closely resemble *R*. *kauffeldi* than they do *R*. *sphenocephala*. We note, however, that the distribution model identified little suitable habitat in the MD-to-VA gap. Our range map is based on confirmed observations, with the expectation that new observations may add to the known range. Our distribution model represents suitable habitat at a finer scale, with coastal impoundments and river valleys standing out and a clear signature of having sampled in part along roads in both the map and the variable importance rankings. The low success rate of the model in predicting presence may reflect local extirpations from still-suitable habitat. Future distribution modeling could use likely absence points instead of random background points, which may allow for more accurate predictions of presence points. The combined use of modeling, interpretation of aerial imagery, and field survey may also help fill these gaps over time.

#### Field identification and genetics

While our study did not identify a definable single morphological or color character for distinguishing *R*. *kauffeldi* from *R*. *sphenocephala*, we did find that a combination of the femoral reticulum, tympanum spots, size and number of dorsal spots, snout shape, and overall coloration provided the correct identification more than nine out of ten times. For now, the primary mating call described by Feinberg et al. [21] remains the only truly diagnostic feature in the field, and our results strongly suggest that the three species (as defined genetically) are reliably distinguishable based on calls.

Characterizing the darkness of the femoral reticulum is critical to identifying *R*. *kauffeldi* correctly, especially in areas of sympatry with *R*. *sphenocephala*. *Rana kauffeldi* always had a reticulum with light spotting on a dark background, although reticula of *R*. *sphenocephala* could have either pattern. Our analysis using ImageJ (S2 Appendix) showed that reticular darkness averaged around 70% for *R*. *kauffeldi* and 55% for *R*. *sphenocephala*, showing that despite the categories used in our analysis, both frogs’ reticula can be more appropriately characterized as “mostly dark.” In most cases, characterizing the reticulum as “mostly dark” or “as much light as dark” will be sufficient. Because this feature is typically hidden when frogs are at rest, identification from photographs of sitting frogs will remain challenging.

Further challenging the correct field identification of non-calling frogs is potential hybridization, particularly between *R*. *kauffeldi* and *R*. *sphenocephala*. Over 10% of the frogs we DNA tested showed apparent admixture based on our small sample of genetic loci. Hybridization has been studied extensively in leopard frogs and documented in the wild for a number of species pairs [64–66]. The small number of genes we had to work with did not allow for detailed genetic analyses beyond simple typing, and may also explain some odd admixtures of *R*. *kauffeldi* with *R*. *pipiens* in the north and *R*. *sphenocephala* in the south. Further research using next-generation sequencing, either with RADseq [67] or target capture [68], would be a logical and important next step in characterizing admixture dynamics of these occasionally syntopic species.

Finally, it is worth noting that the confusion that has plagued leopard frog species delineation in the northeastern U.S. for decades [21] is similar to that in other parts of the range of this taxonomically challenging species complex. For example, starting in the 1970s, the single “*Rana pipiens*” species recognized in Mexico began to be split, based in large part on genetic and call data; approximately 20 species are currently recognized [69,70]. Genetic and call data have similarly shown that the southwestern US contains six members of the leopard species complex, rather than a single entity. Like the US treefrogs *Hyla versicolor* and *H*. *chrysoscelis* [55,71] and some species of *Pseudacris* [14], members of the leopard frog complex, including *R*. *kauffeldi*, appear to be species that cannot always be reliably distinguished without vocalizations. While this may render these taxa challenging for the field biologist, it also emphasizes the importance of follow-up research to document geographic and ecological ranges, regions of species overlap, and conservation needs of these frogs across their ranges.

#### Conservation, management, and information needs

Often when cryptic species are first discovered, little is known about their distribution or conservation status (e.g., [72–74]). As more thorough studies accumulate, and particularly as their ranges and habitat preferences are determined, these taxa are often determined to have very limited distributions and to be of conservation concern (e.g., [75–77]). Often this is because they have small populations, which likely hindered their discovery in the first place. The identification of cryptic species as a special case of new species discovery likewise can yield species of concern, for the simple reason that when a species’ range is subdivided, both resulting ranges and population sizes are by definition reduced [8,13]. For example, several species of genetically distinct leopard frogs in the American Southwest *R*. *pipiens* “complex” [78–80] are now known to be of conservation concern [48,81]. In the southeastern U.S., Pauly et al. [82] determined that a single Threatened salamander species, *Ambystoma* “*cingulatum*,” was in fact two species, one of which (*A*. *bishopi*) was quickly upgraded to Endangered by the U.S. Fish and Wildlife Service [83]. And slender salamanders formerly grouped together as *Batrachoseps attenuatus* may in fact comprise 39 species [84], many of which are globally rare.

In the current situation, *R*. *kauffeldi* overlaps with, and at the northern edge of its range, supplants *R*. *sphenocephala*. It has a far smaller range than *R*. *sphenocephala*, and while it is locally common and likely secure for the moment at the core of its range, it is vulnerable in places. Along with its small range, *R*. *kauffeldi*’s largely coastal distribution is a major reason for conservation concern [21]. Most populations of *R*. *kauffeldi* exist within a highly developed urban and suburban matrix, and the frog’s need for large wetlands (as opposed to *R*. *sphenocephala*, which can occupy small ponds) may render it vulnerable to habitat fragmentation with wetland patches separated by inhospitable dispersal habitat. For species such as leopard frogs that spend considerable time in the uplands, the landscape surrounding the aquatic breeding habitat may also be crucial to long-term persistence [85–87]. Based on typical migration distances reported in the literature on other amphibian species [86], a terrestrial buffer around breeding habitat of several hundred meters is likely necessary to ensure suitable upland habitat, although in at least one highly urban setting the species appears to survive with little surrounding upland greenspace [53]. Studies specific to *R*. *kauffeldi* are needed, including studies to determine genetic connectivity among populations to ensure that the isolated nature of some populations has not led to inbreeding depression [88]. Fragmentation has additional, more immediate direct effects on mobile individuals in the form of road mortality. Highways and other major roads bisect leopard frog habitat throughout the Northeast, and frogs are often killed when crossing them. In fact, many of our samples were obtained from road-killed frogs. High-volume or multi-lane roads may serve as permanent, impenetrable barriers to dispersal, which, along with road mortality, can have considerable impacts on anuran richness and abundance [89,90].

Another conservation consideration is that a small geographic range is associated with greater risk of extinction across taxa and in amphibians specifically [91–94]. *Rana kauffeldi* appears to have the smallest range of any ranid frog on the East Coast, as posited by Feinberg et al. [21]. Of the ranids that range from the mid-Atlantic south to Florida and in some cases west along the Gulf Coast (e.g., *R*. *virgatipes*, *R*. *grylio*, *R*. *capito*), only the extreme endemics *R*. *sevosa* [95] and *R*. *okaloosae* [48] are as restricted as *R*. *kauffeldi*. Apart from the pine barrens treefrog (*Hyla andersoni*) and New Jersey chorus frog (*Pseudacris kalmi*), no eastern US anuran north of Florida has as small a range as *R*. *kauffeldi*. A small range may make a species more susceptible to stochastic events, and for frogs, may exacerbate the impact of fungal pathogens like *Batrachochytrium dendrobatidis* (Bd; [92]). Bd has recently been documented in *R*. *kauffeldi* (JAF, unpublished data) as it has in *R*. *pipiens* and *R*. *sphenocephala* [96,97], and many other ranids [98].

Another point of concern for *R*. *kauffeldi* is the coastal proximity of many populations. Coastal populations of wetland organisms may be threatened by rising sea levels and increasing frequency and intensity of coastal storms, both of which may increase with climate change [99–101]. While the presence of *R*. *kauffeldi* was not strongly tied to climate parameters in our distribution model, the model was not designed to forecast distributional shifts with changing climate or its collateral effects like sea-level rise and storms. Storms may cause saltwater overwash into freshwater habitats, and ongoing research (Feinberg et al., unpubl. data) is addressing the tolerance of *R*. *kauffeldi* to brackish conditions and persistence in coastal sites after major storm events. In Delaware, large calling choruses of *R*. *kauffeldi* disappeared from freshwater impoundments along the Delaware Bay following storms that altered the coastline creating inlets that allowed for inflow of saltwater (J. White, pers. comm.). More inland (typically large-river riparian) populations of *R*. *kauffeldi* may be less vulnerable to these changing coastal conditions, but also possibly less adapted to storm-related flooding.

We recommend additional field inventory, especially during the late winter and early spring calling season when frogs are most easily identified, to clear up remaining uncertainties in the broad-scale distribution of *Rana kauffeldi*. Our understanding of the frog’s distribution at the edges of its range is least well understood, both because range edges are often difficult to characterize and because of the urban landscape of the northern edge. In New York and Connecticut, populations are highly disjunct, in part a function of the heavily developed landscape now dividing surviving populations. In between these populations, there are scattered reports of leopard frogs that are undocumented by recordings or photographs, but our field work did not confirm many of these reports. At the apparent southern edge of the range in North Carolina, the distribution of *R*. *kauffeldi* is just beginning to be understood, and it is not yet clear whether the frog is rare or common in the region and whether the southern range margin is set by physiography, climate, interspecific interactions, or other factors, or extends farther south than our efforts demonstrated. Many states have gaps in local distributional information which, along with an understanding of population vulnerability in habitat patches of different sizes and degrees of urbanization, are a critical need for better understanding the conservation status of *R*. *kauffeldi*.

Finally, we call attention to apparent extirpations of both *R*. *kauffeldi* from coastal regions of the Northeast and *R*. *pipiens*, from an extensive area near the southern edge of its former range. *Rana pipiens* can no longer be found in many locations in PA, NJ, NY, CT, RI, and MA where it once occurred based on museum specimens and historical literature. The recent (April 2017) discovery of a population near Providence, RI (CR, unpublished data) is one bright spot, but this considerable decline certainly warrants greater recognition and concern within the amphibian conservation community. Many extirpations of this species have been documented from western North America [102] and local declines have been noted in the Northeast [26] but widespread extirpation in this region has not previously been reported. Whether extirpations are a result of habitat loss, range shifting with a warming climate, introduced populations failing to sustain themselves, or some other factor is a topic for further research. The apparent northeastern retreat of *R*. *pipiens* echoes the recent finding that another ranid, the Wood Frog, *R*. *sylvatica*, is also retreating northward [103].

### Ecological research agenda for cryptic species and conclusion

Cryptic species remain a fascinating, and rising challenge to systematics, ecology, and conservation, but one that is increasingly met with new research technologies. Much has been written about the problem of identifying cryptic species [8,10,18] and the implications for assessments of species diversity within taxa and globally [104–107]. Less attention has been paid to the follow-up studies necessary to answer basic questions about ecological, behavioral, and morphological differences among species in a cryptic complex. Here we present a framework for answering these important questions in phylogenetics, biogeography, ecology and behavior, morphology, conservation, and implications for scientific literature that should be a standard part of our ecological research agenda following cryptic species discovery (Table 3). This framework differs from one that might reasonably be applied to any newly discovered species in that it focuses on teasing out differences between two or more cryptic species within a complex.

**Table 3:** Research agenda for follow-up studies on a cryptic species discovery, specifically for cases in which one species is split off from another, presumably through genetic analysis.

Information need	Suggested methods
*Phylogenetics and evolution*	
What are the relationships among species within the species complex?	Phylogeny construction
What isolating mechanism facilitated speciation?	Behavioral study, laboratory breeding experiments, biogeographic analysis
*Biogeography*	
Is the range of the newly identified species additional to, or a subset of, the currently mapped range of the species complex?	Field surveys, coupled with any necessary methods of species identification (e.g., genotyping, morphometrics)
Has the species’ range changed due to human land use, and if so, what was it historically?	Determinations of museum specimens
*Ecology and behavior*	
Can species be differentiated by behavior and/or vocalizations?	Behavioral study, bioacoustics
Do the species differ in their ecological roles, including potential provisioning of ecosystem services (or disservices)?	Behavioral and ecological study
*Morphology*	
How can species be differentiated visually in the field or lab?	Morphometrics, photographic analysis
Are there other life stages (e.g., eggs, larvae) or structures (e.g., nests, burrows, webs) needing description?	Examination of museum specimens, field observation, photographic analysis, behavioral study
*Conservation*	
Does the newly-described species have previously unidentified threats?	Threat assessment
Has the species experienced declines?	Examination of museum specimens and field surveys
Is the species of conservation concern?	Conservation status assessment
*Scientific literature*	
What are the impacts of a cryptic species discovery for previously published work on the species complex?	Literature review, meta-analysis combined with biogeographic study

As with any newly described species, there is still much to learn about *R*. *kauffeldi*’s ecology and natural history. Descriptions of *R*. *kauffeldi* egg masses and tadpoles are lacking [108], and the ability to distinguish species based especially on tadpoles would be a boon to detailed natural history, ecological, and demographic studies. For example, adaptations of the various life stages to particular environmental conditions and stressors may help explain the geographic and ecological distributions of the species. Given the highly urban setting of many populations, the susceptibility to environmental contaminants of the various life stages [109], and potential impacts of noise on anuran communication [110], are of great interest. Research into possible competitive interactions may shed light on any niche separation given the overlap with *R*. *sphenocephala* and *R*. *pipiens* and the suggestion of hybridization in both the genetic and morphological data, and help explain observed declines. Finally, additional taxonomic research using a combination of genetics, morphology, and bioacoustics may yet reveal more cryptic diversity within the North American leopard frog complex.

## Supporting information

S1 Appendix Exemplar photographs of characters used in analysis of leopard frog morphology and patterning. (DOCX)

S2 Appendix Reticulum characterization with ImageJ. (DOCX)

S3 Appendix Additional genetic methods and results. (DOCX)

S4 Appendix Morphological and color characters from photographic analysis of 220 leopard frogs (*Rana* spp.) in the eastern U.S. Frogs were identified using genetics and designated as “pure” if they had a 90% or greater match with a single species; individuals with a species match between 10% and 90% were designated as admixed. Sample sizes for each character are given because not every frog had photographs suitable for examination of that character.
(DOCX)

## References

[1] Savage JM. Systematics and the biodiversity crisis. BioScience. 1995;45: 673–679. 10.2307/1312672

[2] Wheeler QD. Systematics, the scientific basis for inventories of biodiversity. Biodivers Conserv. 1995;4: 476–489. 10.1007/BF00056338

[3] Balmford A, Gaston KJ. Why biodiversity surveys are good value. Nature. 1999;398: 204–205. 10.1038/18339

[4] Tracy CR, Brussard PF. Preserving biodiversity: species in landscapes. Ecological Applications. 1994;4: 205–207.

[5] Mace GM. The role of taxonomy in species conservation. Philosophical Transactions of the Royal Society of London B: Biological Sciences. 2004;359: 711–719. 10.1098/rstb.2003.1454 15253356 PMC1693347

[6] Lindenmayer DB, Fischer J, Felton A, Montague-Drake R, D. Manning A, Simberloff D, et al The complementarity of single-species and ecosystem-oriented research in conservation research. Oikos. 2007;116: 1220–1226. 10.1111/j.0030-1299.2007.15683.x

[7] White AM, Zipkin EF, Manley PN, Schlesinger MD. Conservation of avian diversity in the Sierra Nevada: Moving beyond a single-species management focus. PLoS ONE. 2013;8: e63088 10.1371/journal.pone.0063088 23667579 PMC3646733

[8] Bickford D, Lohman DJ, Sodhi NS, Ng PKL, Meier R, Winker K, et al Cryptic species as a window on diversity and conservation. Trends in Ecology & Evolution. 2007;22: 148–155. 10.1016/j.tree.2006.11.004 17129636

[9] Forti LR, Lingnau R, Encarnação LC, Bertoluci J, Toledo LF. Can treefrog phylogeographical clades and species’ phylogenetic topologies be recovered by bioacoustical analyses? PLOS ONE. 2017;12: e0169911 10.1371/journal.pone.0169911 28235089 PMC5325193

[10] Angulo A, Icochea J. Cryptic species complexes, widespread species and conservation: Lessons from Amazonian frogs of the Leptodactylus marmoratus group (Anura: Leptodactylidae). Systematics and Biodiversity. 2010;8: 357–370. 10.1080/14772000.2010.507264

[11] Gehara M, Canedo C, Haddad CFB, Vences M. From widespread to microendemic: molecular and acoustic analyses show that Ischnocnema guentheri (Amphibia: Brachycephalidae) is endemic to Rio de Janeiro, Brazil. Conserv Genet. 2013;14: 973–982. 10.1007/s10592-013-0488-5

[12] Rodríguez A, Dugo-Cota Á, Montero-Mendieta S, Gonzalez-Voyer A, Bosch RA, Vences M, et al Cryptic within cryptic: genetics, morphometrics, and bioacoustics delimitate a new species of Eleutherodactylus (Anura: Eleutherodactylidae) from Eastern Cuba. Zootaxa. 2017;4221: 501–552. doi: 10.11646/zootaxa.4221.5.1 28187641 10.11646/zootaxa.4221.5.1

[13] Lemmon EM, Lemmon AR, Collins JT, Lee-Yaw JA, Cannatella DC. Phylogeny-based delimitation of species boundaries and contact zones in the trilling chorus frogs (Pseudacris). Molecular phylogenetics and evolution. 2007;44: 1068–1082. 10.1016/j.ympev.2007.04.010 17562372

[14] Lemmon EM, Lemmon AR, Collins JT, Cannatella DC. A new North American chorus frog species (Pseudacris: Hylidae: Amphibia) from the south-central United States. Zootaxa. 2008;1675: 1–30.

[15] Corser JD, Roblee KJ, Johnson G. Shifting status and distribution of range margin chorus frog (Pseudacris) populations in eastern Great Lakes watersheds. Journal of Great Lakes Research. 2012;38: 806–811. 10.1016/j.jglr.2012.09.005

[16] Seburn DC, Gunson K, Schueler FW. Apparent widespread decline of the boreal chorus frog (Pseudacris maculata) in eastern Ottawa. The Canadian Field-Naturalist. 2014;128: 151–157.

[17] Niemiller ML, Near TJ, Fitzpatrick BM. Delimiting species using multilocus data: diagnosing cryptic diversity in the southern cavefish, Typhlichthys subterraneus (Teleostei: Amblyopsidae). Evolution. 2012;66: 846–866. 10.1111/j.1558-5646.2011.01480.x 22380444

[18] Niemiller ML, Graening GO, Fenolio DB, Godwin JC, Cooley JR, Pearson WD, et al Doomed before they are described? The need for conservation assessments of cryptic species complexes using an amblyopsid cavefish (Amblyopsidae: Typhlichthys) as a case study. Biodivers Conserv. 2013;22: 1799–1820. 10.1007/s10531-013-0514-4

[19] Trillo PA, Narvaez AE, Ron SR, Hoke KL. Mating patterns and post-mating isolation in three cryptic species of the Engystomops petersi species complex. PLOS ONE. 2017;12: e0174743 10.1371/journal.pone.0174743 28388628 PMC5384746

[20] Funk WC, Caminer M, Ron SR. High levels of cryptic species diversity uncovered in Amazonian frogs. Proc R Soc B. 2011;279: 1806–1814. 10.1098/rspb.2011.1653 22130600 PMC3297442

[21] Feinberg JA, Newman CE, Watkins-Colwell GJ, Schlesinger MD, Zarate B, Curry BR, et al Cryptic diversity in Metropolis: Confirmation of a new leopard frog species (Anura: Ranidae) from New York City and surrounding Atlantic Coast regions. PloS one. 2014;9: e108213 10.1371/journal.pone.0108213 25354068 PMC4212910

[22] Yuan Z-Y, Zhou W-W, Chen X, Poyarkov NA, Chen H-M, Jang-Liaw N-H, et al Spatiotemporal diversification of the true frogs (genus Rana): A historical framework for a widely studied group of model organisms. Syst Biol. 2016;65: 824–842. 10.1093/sysbio/syw055 27288482

[23] Newman CE, Feinberg JA, Rissler LJ, Burger J, Shaffer HB. A new species of leopard frog (Anura: Ranidae) from the urban northeastern US. Molecular Phylogenetics and Evolution. 2012;63: 445–455. 10.1016/j.ympev.2012.01.021 22321689 PMC4135705

[24] Kauffeld CF. New York the type locality of Rana pipiens Schreber. Herpetologica. 1936;1: 11.

[25] Kauffeld CF. The status of the leopard frogs, Rana brachycephala and Rana pipiens. Herpetologica. 1937;1: 84–87.

[26] Klemens MW. Amphibians and reptiles of Connecticut and adjacent regions State Geological and Natural History Survey of Connecticut Hartford, USA; 1993.

[27] Pace AE. Systematic and biological studies of the leopard frogs (Rana pipiens complex) of the United States Miscellaneous Publications, Museum of Zoology, University of Michigan, No148. 1974; 140.

[28] Moore JA. Geographic variation in Rana pipiens Schreber of eastern North America. Bull Am Mus Nat Hist. 1944;82: 345–370. 10.2307/2405448

[29] Porter KR. Diploid and androgenetic haploid hybridization between two forms of Rana pipiens, Schreber. Biol Bull. 1941;80: 238–264. 10.2307/1537601

[30] ESRI. ArcGIS Desktop: Release 10.3 Redlands, CA: Environmental Systems Research Institute; 2014.

[31] Kearse M, Moir R, Wilson A, Stones-Havas S, Cheung M, Sturrock S, et al Geneious Basic: An integrated and extendable desktop software platform for the organization and analysis of sequence data. Bioinformatics. 2012;28: 1647–1649. 10.1093/bioinformatics/bts199 22543367 PMC3371832

[32] Pritchard JK, Stephens M, Donnelly P. Inference of population structure using multilocus genotype data. Genetics. 2000;155: 945–959. 10835412 10.1093/genetics/155.2.945 PMC1461096

[33] Falush D, Stephens M, Pritchard JK. Inference of population structure using multilocus genotype data: Linked loci and correlated allele frequencies. Genetics. 2003;164: 1567–1587. 12930761 10.1093/genetics/164.4.1567 PMC1462648

[34] Stephens M, Smith NJ, Donnelly P. A new statistical method for haplotype reconstruction from population data. The American Journal of Human Genetics. 2001;68: 978–989. 10.1086/319501 11254454 PMC1275651

[35] Stephens M, Donnelly P. A comparison of Bayesian methods for haplotype reconstruction from population genotype data. The American Journal of Human Genetics. 2003;73: 1162–1169. 10.1086/379378 14574645 PMC1180495

[36] Earl DA, vonHoldt BM. STRUCTURE HARVESTER: a website and program for visualizing STRUCTURE output and implementing the Evanno method. Conservation Genet Resour. 2012;4: 359–361. 10.1007/s12686-011-9548-7

[37] Wandeler P, Hoeck PEA, Keller LF. Back to the future: museum specimens in population genetics. Trends in Ecology & Evolution. 2007;22: 634–642. 10.1016/j.tree.2007.08.017 17988758

[38] R Development Core Team. R: A language and environment for statistical computing [Internet]. Vienna, Austria: R Foundation for Statistical Computing; 2013 Available: http://www.R-project.org

[39] Liaw A, Wiener M. Classification and regression by randomForest. R News. 2002;2: 18–22.

[40] Breiman L. Random forests. Machine Learning. 2001;45: 5–32.

[41] Strobl C, Boulesteix AL, Kneib T, Augustin T, Zeileis A. Conditional variable importance for random forests. BMC Bioinformatics. 2008;9: 307 10.1186/1471-2105-9-307 18620558 PMC2491635

[42] IUCN, Conservation International, and NatureServe. Amphibian range maps The IUCN Red List of Threatened Species [Internet]. 2013 Available: http://www.iucnredlist.org

[43] Howard TG, Schlesinger MD. Wildlife habitat connectivity in the changing climate of New York’s Hudson Valley. Annals of the New York Academy of Sciences. 2013;1298: 103–109. 10.1111/nyas.12172 23777545

[44] USGS and USDA. Federal standards and procedures for the National Watershed Boundary Dataset (WBD) (4 ed.): U.S. Geological Survey Techniques and Methods 11–A3 [Internet]. U.S. Geological Survey and U.S. Department of Agriculture, Natural Resources Conservation Service; 2013. Available: http://pubs.usgs.gov/tm/tm11a3/

[45] Master LL, Faber-Langendoen D, Bittman R, Hammerson G, Heidel B, Ramsay L, et al NatureServe conservation status assessments: Factors for evaluating species and ecosystem risk Arlington, VA: NatureServe; 2012.

[46] Faber-Langendoen D, Nichols J, Master L, Snow K, Tomaino A, Bittman R, et al NatureServe conservation status assessments: methodology for assigning ranks Arlington, Virginia: NatureServe; 2012 6 p. 44.

[47] NatureServe. NatureServe Explorer: an online encyclopedia of life [web application] Version 7.1 [Internet]. 2016 [cited 1 Feb 2016]. Available: http://www.natureserve.org/explorer

[48] Lannoo MJ. Amphibian declines: The conservation status of United States species University of California Press; 2005.

[49] Taylor B, Skelly D, Demarchis LK, Slade MD, Galusha D, Rabinowitz PM. Proximity to pollution sources and risk of amphibian limb malformation. Environmental Health Perspectives. 2005;113: 1497–1501. 10.1289/ehp.7585 16263502 PMC1310909

[50] Egea-Serrano A, Relyea RA, Tejedo M, Torralva M. Understanding of the impact of chemicals on amphibians: a meta-analytic review. Ecology and Evolution. 2012;2: 1382–1397. 10.1002/ece3.249 22957147 PMC3434931

[51] Feinberg JA, Kiviat E, Schlesinger MD, Burger J. A rapid assessment of post-hurricane impacts on populations of the new leopard frog species, Rana (Lithobates) kauffeldi sp. nov., in the New York City metropolitan region. unpublshed manuscript;

[52] NatureServe. NatureServe conservation status assessments: Rank calculator version 3.186 [Internet]. Arlington, VA: NatureServe; 2015 Available: http://connect.natureserve.org/publications/StatusAssess_RankCalculator

[53] Kiviat E. Frog call surveys in an urban wetland complex, the Hackensack Meadowlands, New Jersey, 2006. Urban Habitats. 2011;6.

[54] The Mid-Atlantic Center for Herpetology and Conservation. Pennsylvania Amphibian & Reptile Survey [Internet]. 2016 [cited 19 Dec 2016]. Available: http://paherpsurvey.org/

[55] Conant R, Collins JT. A field guide to reptiles & amphibians: Eastern and central North America. Houghton Mifflin Harcourt; 1998.

[56] Klemens MW, Kiviat E, Schmidt RE. Distribution of the northern leopard frog (Rana pipiens), in the Lower Hudson and Housatonic River Valleys. Northeastern Environmental Science. 1987;6: 99–101.

[57] Florida R, Gulden T, Mellander C. The rise of the mega-region. Cambridge J Regions Econ Soc. 2008;1: 459–476. 10.1093/cjres/rsn018

[58] Fenneman NM, Johnson DW. Physical divisions of the United States [Internet]. Washington D.C: U.S. Geological Survey; 1946 Available: https://water.usgs.gov/GIS/metadata/usgswrd/XML/physio.xml

[59] Fenneman NM. Physiography of eastern United States New York: McGraw-Hill Book Company; 1938.

[60] Noss R. Announcing the world’s 36th biodiversity hotspot: The North American Coastal Plain [Internet]. 18 2 2016 [cited 15 Dec 2016]. Available: http://www.cepf.net/news/top_stories/Pages/Announcing-the-Worlds-36th-Biodiversity-Hotspot.aspx#.WFL53PS3lWI

[61] Bailey RG. Ecoregions: The ecosystem geography of the oceans and continents New York: Springer-Verlag; 1998.

[62] The Nature Conservancy. Mid-Atlantic Coastal Plain ecoregional plan Durham, NC: The Nature Conservancy; 2001.

[63] Ray N, Adams JM. A GIS-based vegetation map of the world at the Last Glacial Maximum (25,000–15,000 BP). Internet Archaeology. 2001; doi: 10.11141/ia.11.2

[64] Platz JE. Sympatric interaction between two forms of leopard frog (Rana pipiens complex) in Texas. Copeia. 1972;1972: 232–240. 10.2307/1442482

[65] Platz JE, Frost JS. Rana yavapaiensis, a new species of leopard frog (Rana pipiens complex). Copeia. 1984;1984: 940–948. 10.2307/1445338

[66] Parris MJ. Hybridization in leopard frogs (Rana pipiens complex): Variation in interspecific hybrid larval fitness components along a natural contact zone. Evol Ecol Res. 2001;3: 107–116.

[67] Andrews KR, Good JM, Miller MR, Luikart G, Hohenlohe PA. Harnessing the power of RADseq for ecological and evolutionary genomics. Nat Rev Genet. 2016;17: 81–92. 10.1038/nrg.2015.28 26729255 PMC4823021

[68] Jones MR, Good JM. Targeted capture in evolutionary and ecological genomics. Mol Ecol. 2016;25: 185–202. 10.1111/mec.13304 26137993 PMC4823023

[69] Hillis DM. Systematics of the Rana pipiens complex: puzzle and paradigm. Annu Rev Ecol Syst. 1988;19: 39–63. 10.1146/annurev.ecolsys.19.1.39

[70] AmphibiaWeb. University of California, Berkeley, CA, USA [Internet]. 2018 [cited 7 Aug 2018]. Available: https://amphibiaweb.org

[71] Johnson C. Species recognition in Hyla versicolor complex. Texas Journal of Science. 1966;18: 361.

[72] Esselstyn JA, Achmadi AS, Maharadatunkamsi. A new species of shrew (Soricomorpha: Crocidura) from West Java, Indonesia. Journal of Mammalogy. 2014;95: 216–224. 10.1644/13-MAMM-A-215

[73] Brown RM. A new species of stream frog of the genus Hylarana from the mountains of southern Mindanao Island, Philippines. Herpetologica. 2015;71: 223–233. 10.1655/Herpetologica-D-14-00075

[74] Howlader MSA, Nair A, Merilä J. A new species of frog (Anura: Dicroglossidae) discovered from the mega city of Dhaka. PLOS ONE. 2016;11: e0149597 10.1371/journal.pone.0149597 26934699 PMC4801011

[75] Jones T, Ehardt CL, Butynski TM, Davenport TRB, Mpunga NE, Machaga SJ, et al The highland mangabey Lophocebus kipunji: A new species of African monkey. Science. 2005;308: 1161–1164. 10.1126/science.1109191 15905399

[76] Venchi A, Wilson SK, Borsboom AC. A new blind snake (Serpentes: Typhlopidae) from an endangered habitat in south-eastern Queensland, Australia. Zootaxa. 2015;3990: 272–278. doi: 10.11646/zootaxa.3990.2.7 26250233 10.11646/zootaxa.3990.2.7

[77] Clulow S, Anstis M, Keogh JS, Catullo RA. A new species of Australian frog (Myobatrachidae: Uperoleia) from the New South Wales mid-north coast sandplains. Zootaxa. 2016;4184: 285–315. doi: 10.11646/zootaxa.4184.2.3 27811640 10.11646/zootaxa.4184.2.3

[78] Frost JS, Bagnara JT. A new species of leopard frog (Rana pipiens complex) from northwestern Mexico. Copeia. 1976;1976: 332–338. 10.2307/1443955

[79] Platz JE, Mecham JS. Rana chiricahuensis, a New Species of Leopard Frog (Rana pipiens Complex) from Arizona. Copeia. 1979;1979: 383–390. 10.2307/1443211

[80] Platz JE. Rana subaquavocalis, a remarkable new species of leopard frog (Rana pipiens complex) from southeastern Arizona that calls under water. Journal of Herpetology. 1993;27: 154–162. 10.2307/1564931

[81] Clarkson RW, Rorabaugh JC. Status of leopard frogs (Rana pipiens complex: Ranidae) in Arizona and southeastern California. The Southwestern Naturalist. 1989;34: 531–538. 10.2307/3671513

[82] Pauly GB, Piskurek O, Shaffer HB. Phylogeographic concordance in the southeastern United States: the flatwoods salamander, Ambystoma cingulatum, as a test case. Molecular Ecology. 2007;16: 415–429. 10.1111/j.1365-294X.2006.03149.x 17217354

[83] United States Fish and Wildlife Service. Endangered and threatened wildlife and plants; determination of endangered status for reticulated flatwoods salamander; designation of critical habitat for frosted flatwoods salamander and reticulated flatwoods salamander; Final rule. Federal Register. 2009;74: 6699–6774.

[84] Highton R. Detecting cryptic species in phylogeographic studies: Speciation in the California Slender Salamander, Batrachoseps attenuatus. Molecular Phylogenetics and Evolution. 2014;71: 127–141. 10.1016/j.ympev.2013.11.002 24269595

[85] Pope SE, Fahrig L, Merriam HG. Landscape complementation and metapopulation effects on leopard frog populations. Ecology. 2000;81: 2498–2508.

[86] Semlitsch RD, Bodie JR. Biological criteria for buffer zones around wetlands and riparian habitats for amphibians and reptiles. ConservBiol. 2003;17: 1219–1228.

[87] Searcy CA, Gabbai-Saldate E, Bradley Shaffer H. Microhabitat use and migration distance of an endangered grassland amphibian. Biological Conservation. 2013;158: 80–87. 10.1016/j.biocon.2012.08.033

[88] Franklin IR. Evolutionary change in small populations In: Soule ME, Wilcox BA, editors. Conservation biology: An evolutionary-ecological perspective. Sunderland, MA: Sinauer Associates; 1980 pp. 135–139.

[89] Cosentino BJ, Marsh DM, Jones KS, Apodaca JJ, Bates C, Beach J, et al Citizen science reveals widespread negative effects of roads on amphibian distributions. Biological Conservation. 2014;180: 31–38. 10.1016/j.biocon.2014.09.027

[90] Marsh DM, Cosentino BJ, Jones KS, Apodaca JJ, Beard KH, Bell JM, et al Effects of roads and land use on frog distributions across spatial scales and regions in the Eastern and Central United States. Diversity Distrib. 2017;23: 158–170. 10.1111/ddi.12516

[91] Purvis A, Gittleman JL, Cowlishaw G, Mace GM. Predicting extinction risk in declining species. Proceedings of the Royal Society of London B: Biological Sciences. 2000;267: 1947–1952. 10.1098/rspb.2000.1234 11075706 PMC1690772

[92] Bielby J, Cooper N, Cunningham A a., Garner T w. j., Purvis A. Predicting susceptibility to future declines in the world’s frogs. Conservation Letters. 2008;1: 82–90. 10.1111/j.1755-263X.2008.00015.x

[93] Cooper N, Bielby J, Thomas GH, Purvis A. Macroecology and extinction risk correlates of frogs. Global Ecology and Biogeography. 2008;17: 211–221. 10.1111/j.1466-8238.2007.00355.x

[94] Sodhi NS, Bickford D, Diesmos AC, Lee TM, Koh LP, Brook BW, et al Measuring the meltdown: Drivers of global amphibian extinction and decline. PLOS ONE. 2008;3: e1636 10.1371/journal.pone.0001636 18286193 PMC2238793

[95] Richter SC, Jensen JB. Rana sevosa Lannoo M, ed Amphibian declines: The conservation status of United States species. University of California Press; 2005.

[96] Voordouw MJ, Adama D, Houston B, Govindarajulu P, Robinson J. Prevalence of the pathogenic chytrid fungus, Batrachochytrium dendrobatidis, in an endangered population of northern leopard frogs, Rana pipiens. BMC Ecology. 2010;10: 6 10.1186/1472-6785-10-6 20202208 PMC2846871

[97] Chatfield MWH, Brannelly LA, Robak MJ, Freeborn L, Lailvaux SP, Richards-Zawacki CL. Fitness consequences of infection by Batrachochytrium dendrobatidis in northern leopard frogs (Lithobates pipiens). EcoHealth. 2013;10: 90–98. 10.1007/s10393-013-0833-7 23604643

[98] Olson DH, Aanensen DM, Ronnenberg KL, Powell CI, Walker SF, Bielby J, et al Mapping the Global Emergence of Batrachochytrium dendrobatidis, the Amphibian Chytrid Fungus. PLOS ONE. 2013;8: e56802 10.1371/journal.pone.0056802 23463502 PMC3584086

[99] Scavia D, Field JC, Boesch DF, Buddemeier RW, Burkett V, Cayan DR, et al Climate change impacts on U.S. coastal and marine ecosystems. Estuaries. 2002;25: 149–164.

[100] Hopkinson CS, Lugo AE, Alber M, Covich AP, Van Bloem SJ. Forecasting effects of sea-level rise and windstorms on coastal and inland ecosystems. Frontiers in Ecology and the Environment. 2008;6: 255–263.

[101] Pacifici M, Foden WB, Visconti P, Watson JEM, Butchart SHM, Kovacs KM, et al Assessing species vulnerability to climate change. Nature Clim Change. 2015;5: 215–224. 10.1038/nclimate2448

[102] Rorabaugh JC. Rana pipiens Lannoo M, ed Amphibian declines: The conservation status of United States species. University of California Press; 2005.

[103] Amburgey SM, Miller DAW, Campbell Grant EH, Rittenhouse TAG, Benard MF, Richardson JL, et al Range position and climate sensitivity: The structure of among-population demographic responses to climatic variation. Glob Change Biol. 2017; 10.1111/gcb.13817 28833972

[104] Pfenninger M, Schwenk K. Cryptic animal species are homogeneously distributed among taxa and biogeographical regions. BMC Evolutionary Biology. 2007;7: 121 10.1186/1471-2148-7-121 17640383 PMC1939701

[105] Trontelj P, Fišer C. Cryptic species diversity should not be trivialised. Systematics and Biodiversity. 2009;7: 1–3.

[106] Scheffers BR, Joppa LN, Pimm SL, Laurance WF. What we know and don’t know about Earth’s missing biodiversity. Trends in Ecology & Evolution. 2012;27: 501–510. 10.1016/j.tree.2012.05.008 22784409

[107] León GP-P de, Poulin R. Taxonomic distribution of cryptic diversity among metazoans: not so homogeneous after all. Biology Letters. 2016;12: 20160371 10.1098/rsbl.2016.0371 27555648 PMC5014032

[108] Altig R, McDiarmid RW. Handbook of larval amphibians of the United States and Canada Cornell University Press; 2015.

[109] Lambert MR, Giller GSJ, Barber LB, Fitzgerald KC, Skelly DK. Suburbanization, estrogen contamination, and sex ratio in wild amphibian populations. PNAS. 2015;112: 11881–11886. 10.1073/pnas.1501065112 26372955 PMC4586825

[110] Shannon G, McKenna MF, Angeloni LM, Crooks KR, Fristrup KM, Brown E, et al A synthesis of two decades of research documenting the effects of noise on wildlife. Biol Rev. 2016;91: 982–1005. 10.1111/brv.12207 26118691

