# Supplementary material for: Follow-up ecological studies for cryptic species discoveries: Decrypting the leopard frogs of the eastern U.S
Source: PLoS One. 2018 Nov 9;13(11):e0205805. doi: 10.1371/journal.pone.0205805 (PMC6226167; doi:10.1371/journal.pone.0205805)
Supplement: S1 Appendix — (DOCX) [file pone.0205805.s001.docx]

S1 Appendix. Exemplar photographs of characters used in analysis of leopard frog morphology and patterning.

| **Dorsal spotting** | | | | **Snout shape** |
| --- | --- | --- | --- | --- |
| 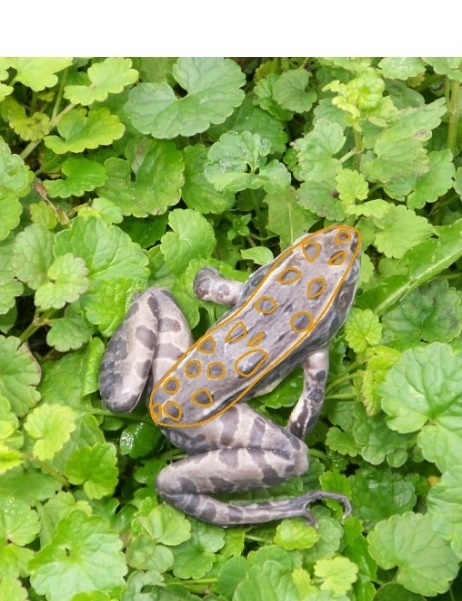 | 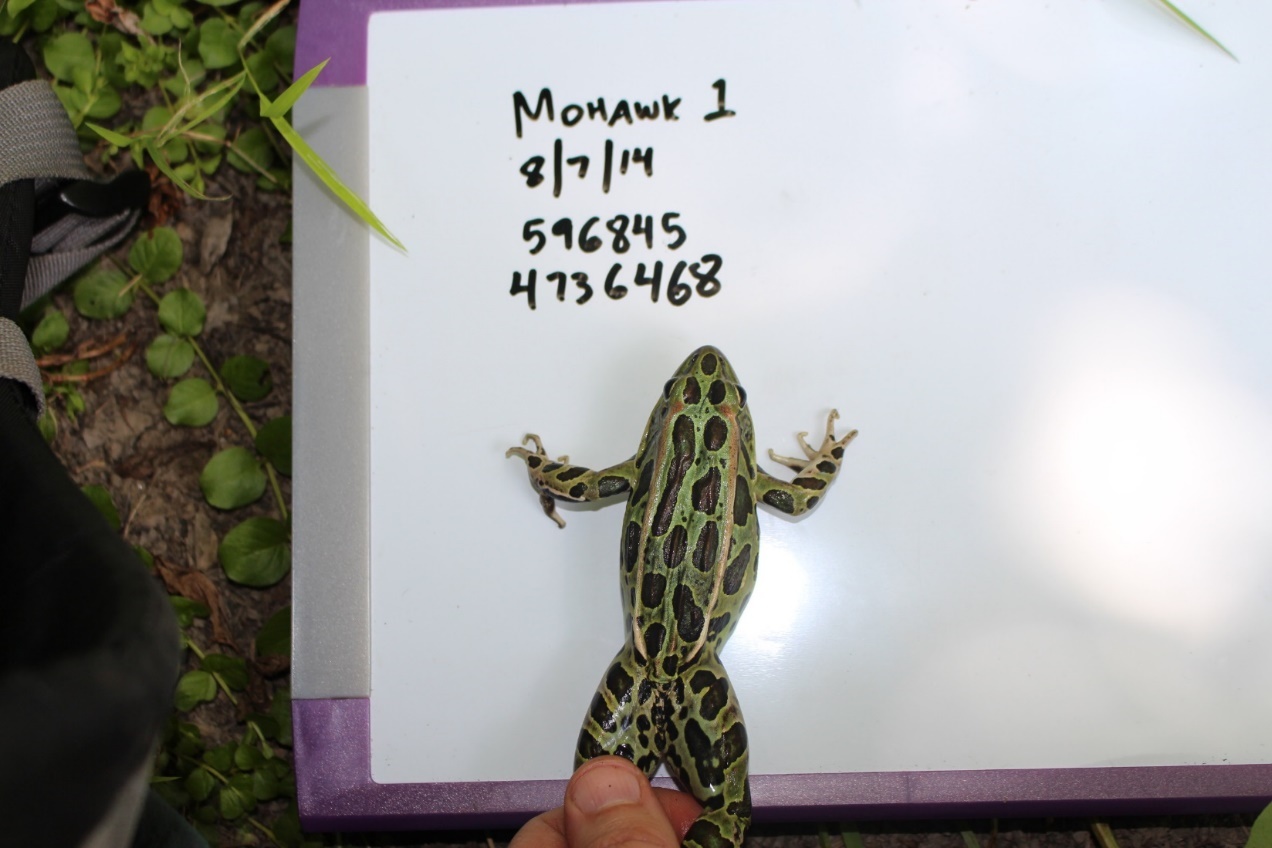 | 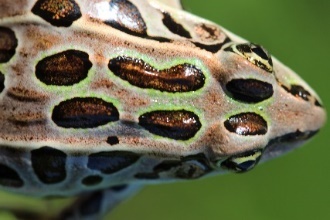 | 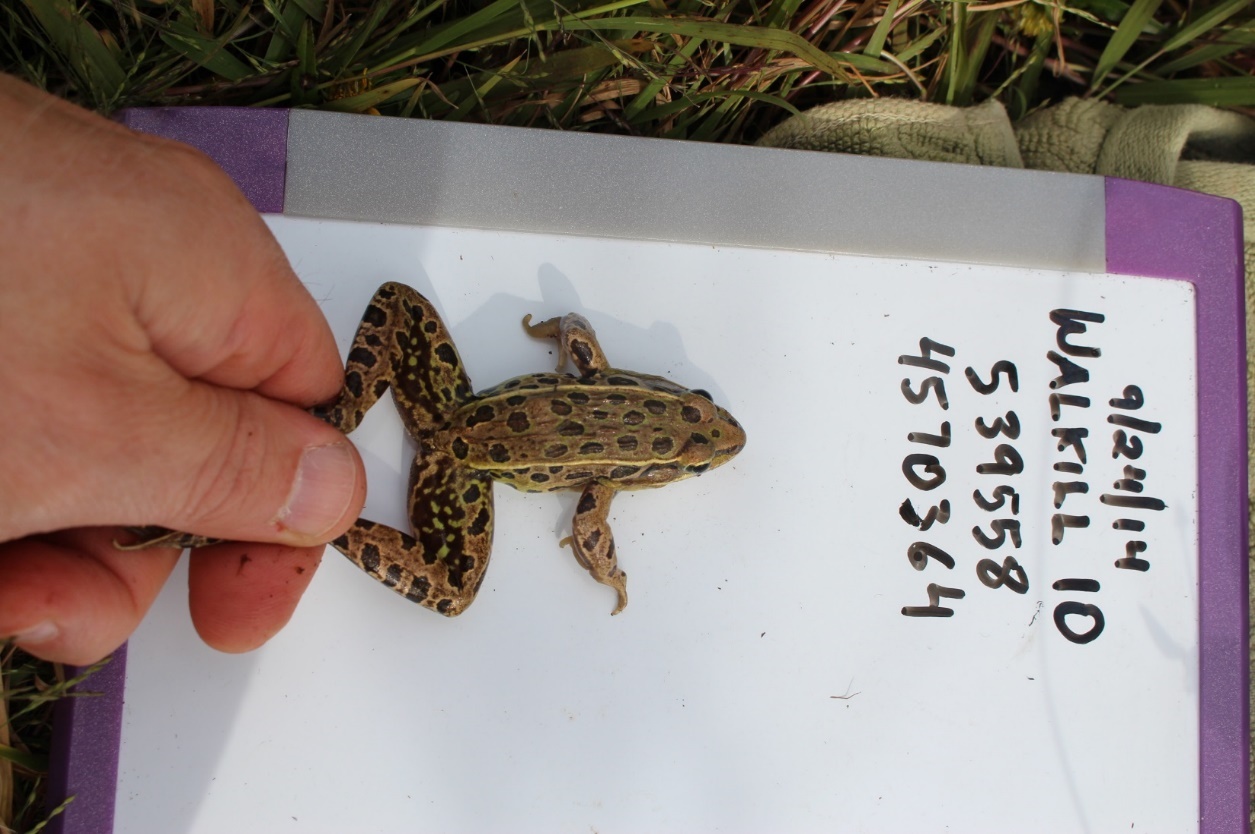 | 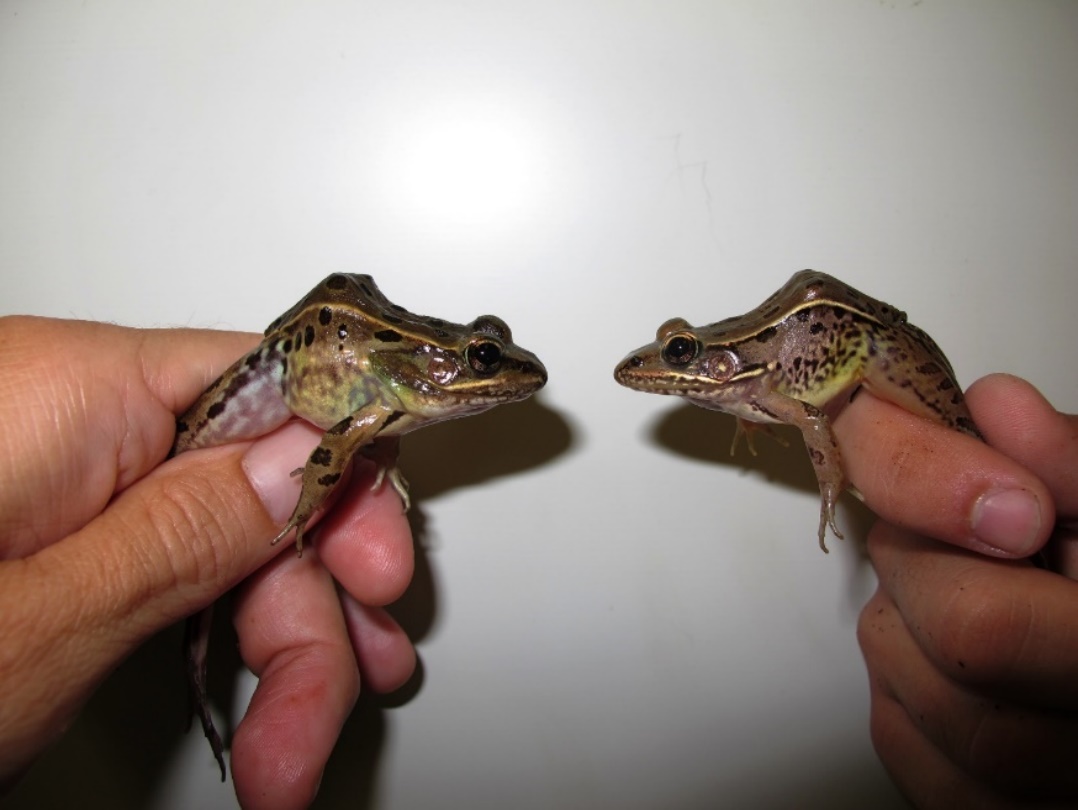 |
| Area of dorsum within which to count spots: between dorsolateral folds and from snout to vent, outlined in orange. | Large snout spot, spots larger than or equal to eye | Snout spot absent, spots larger than or equal eye | Small snout spot, spots smaller than eye | Blunt (left), pointed (right) |
| **Tympanum spots** | | |  | |
| 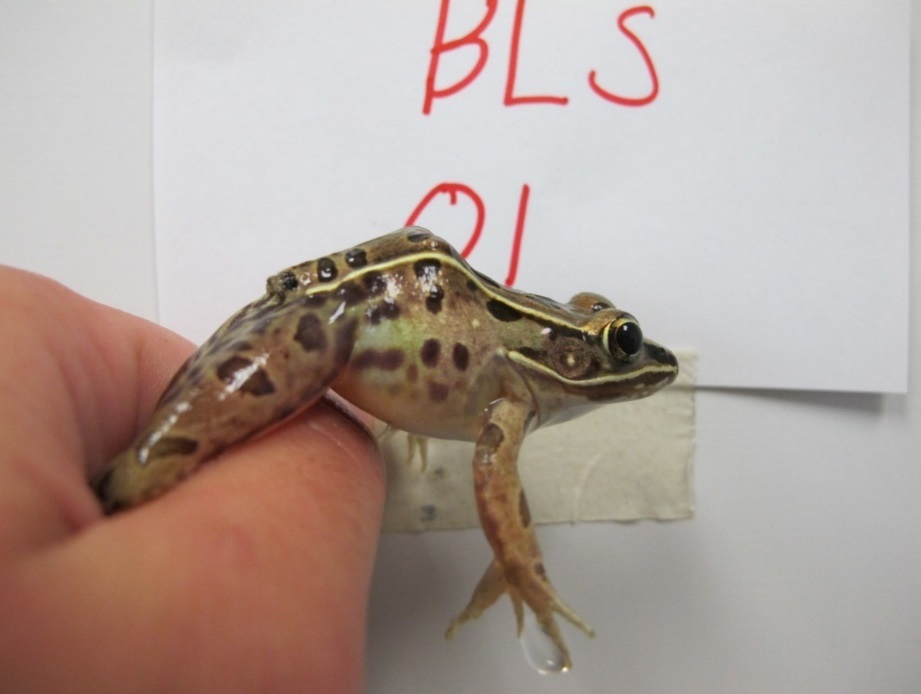 | 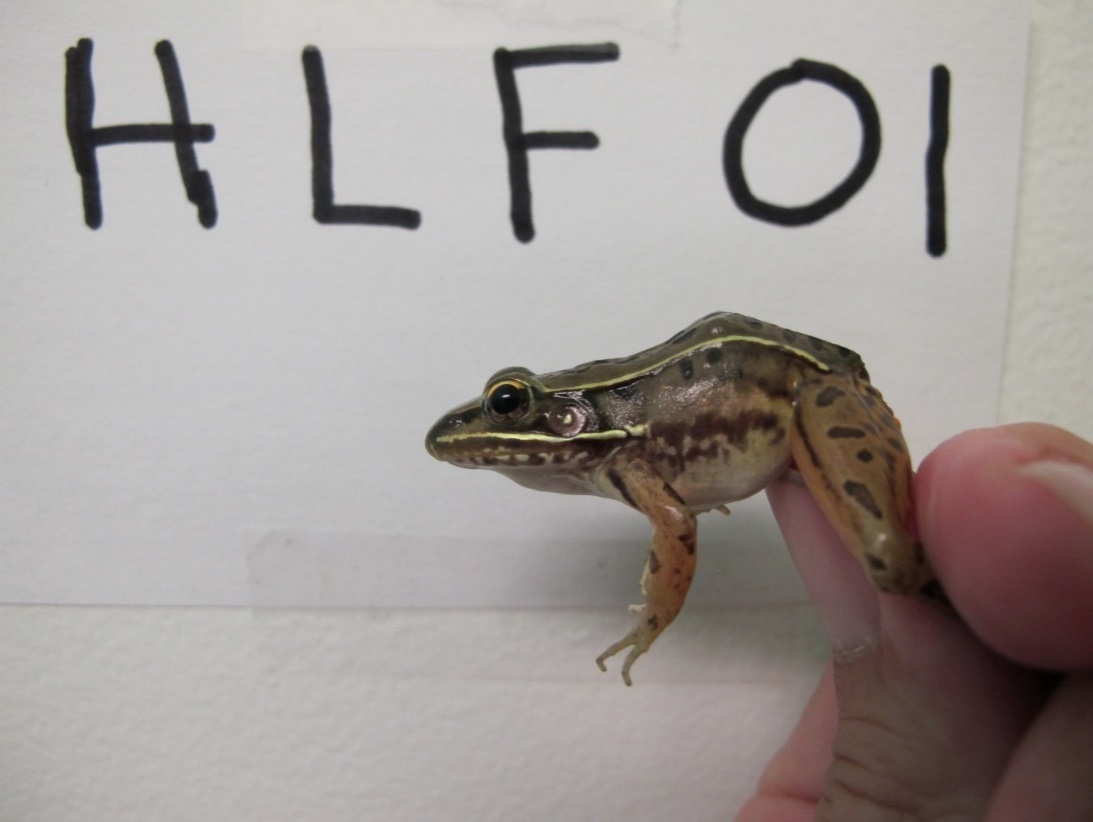 | 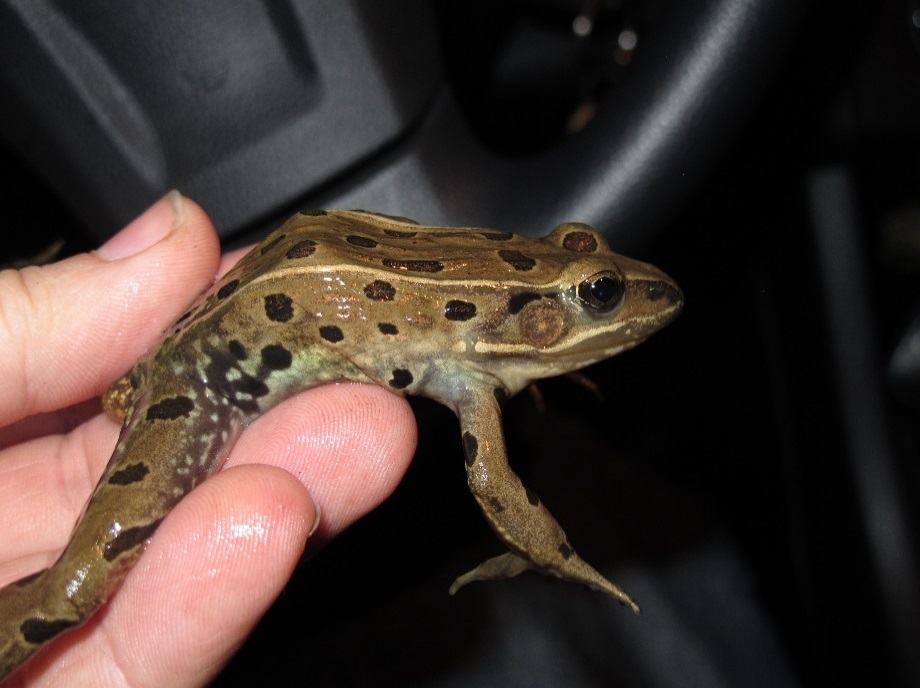 | 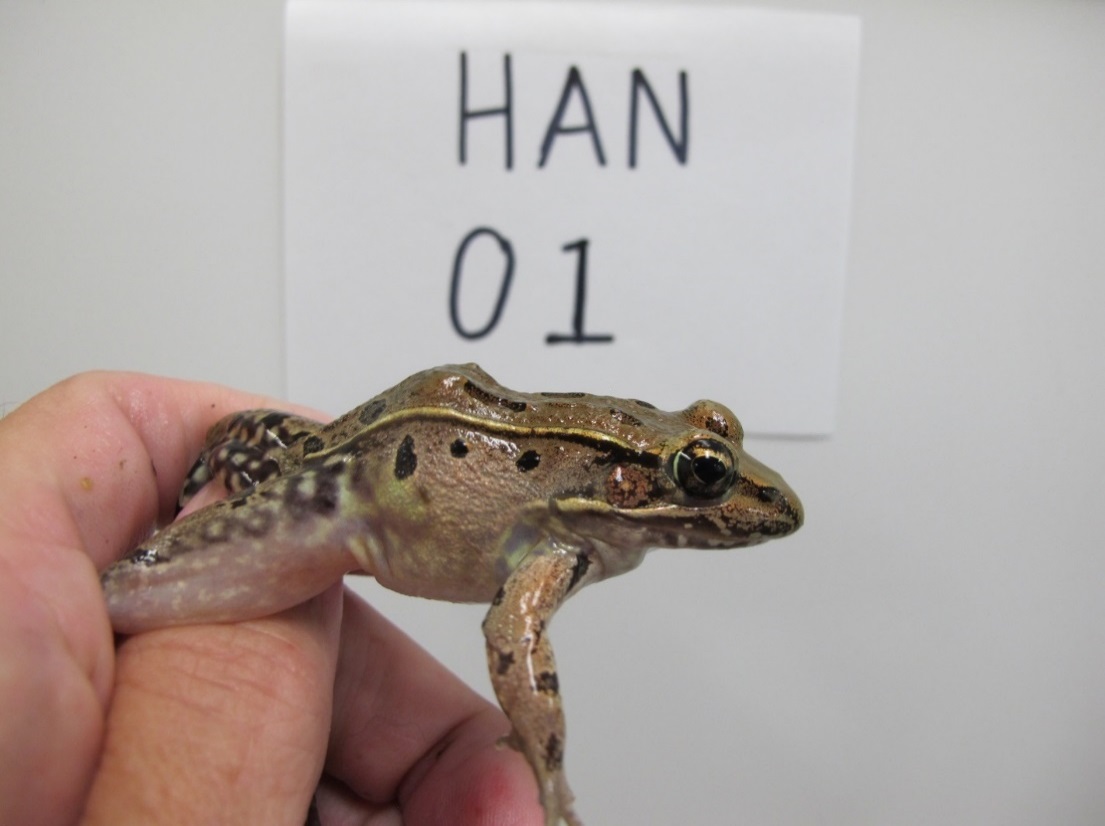 | 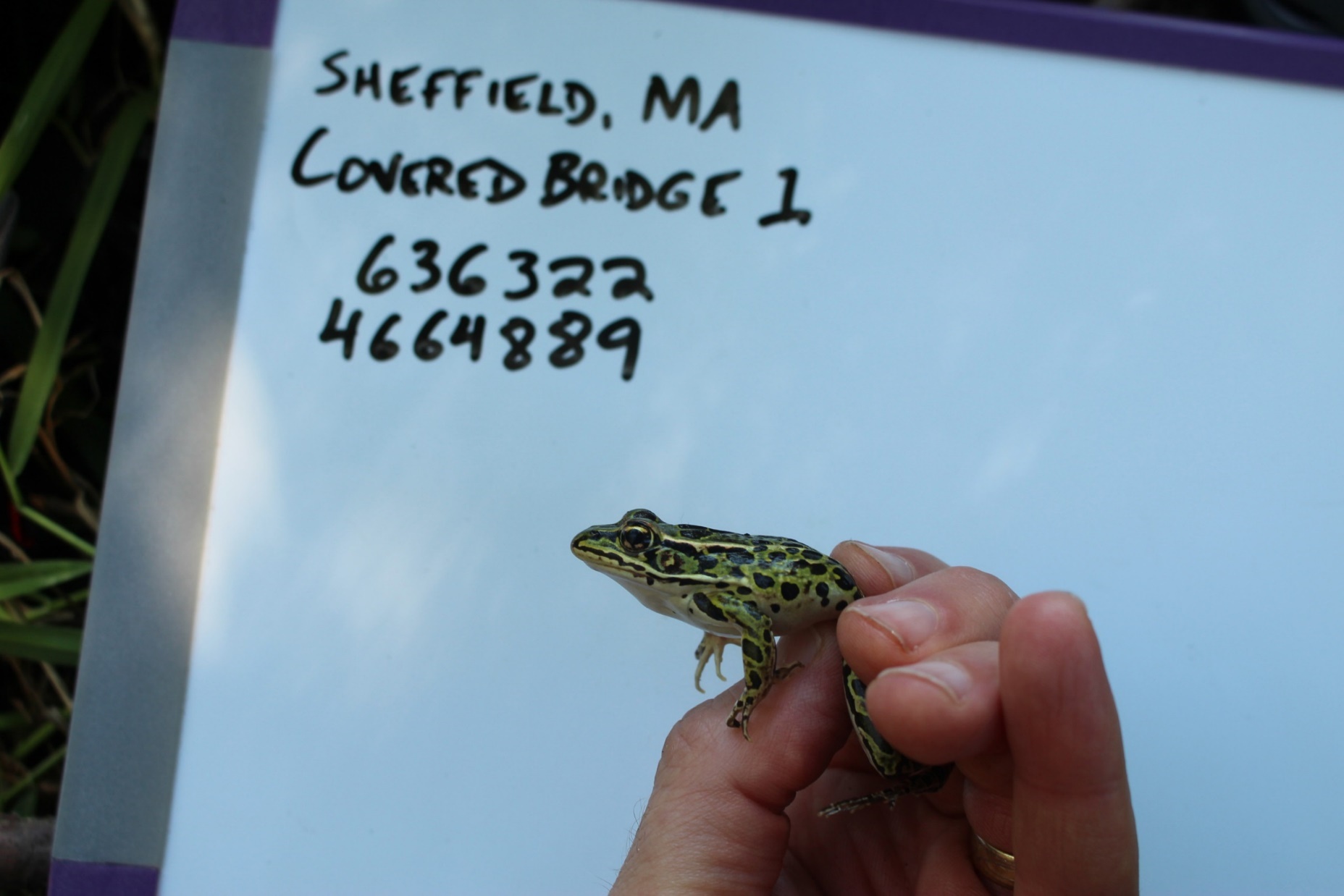 |
| Sharp dot, white/cream | Sharp blotch, white/cream | Present; indistinct, white/cream | Present; indistinct, white/cream | Sharp blotch, green |
| 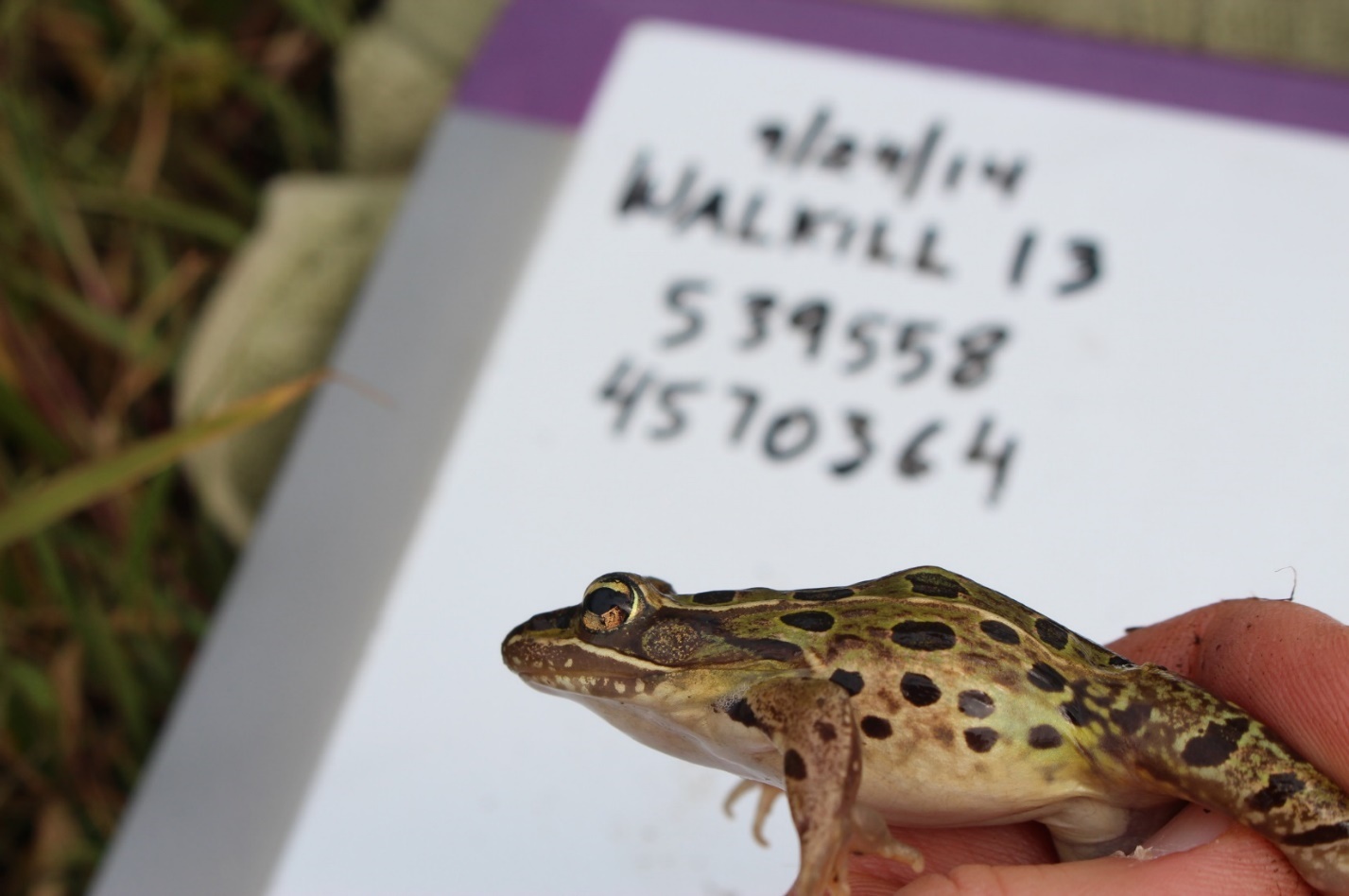 | 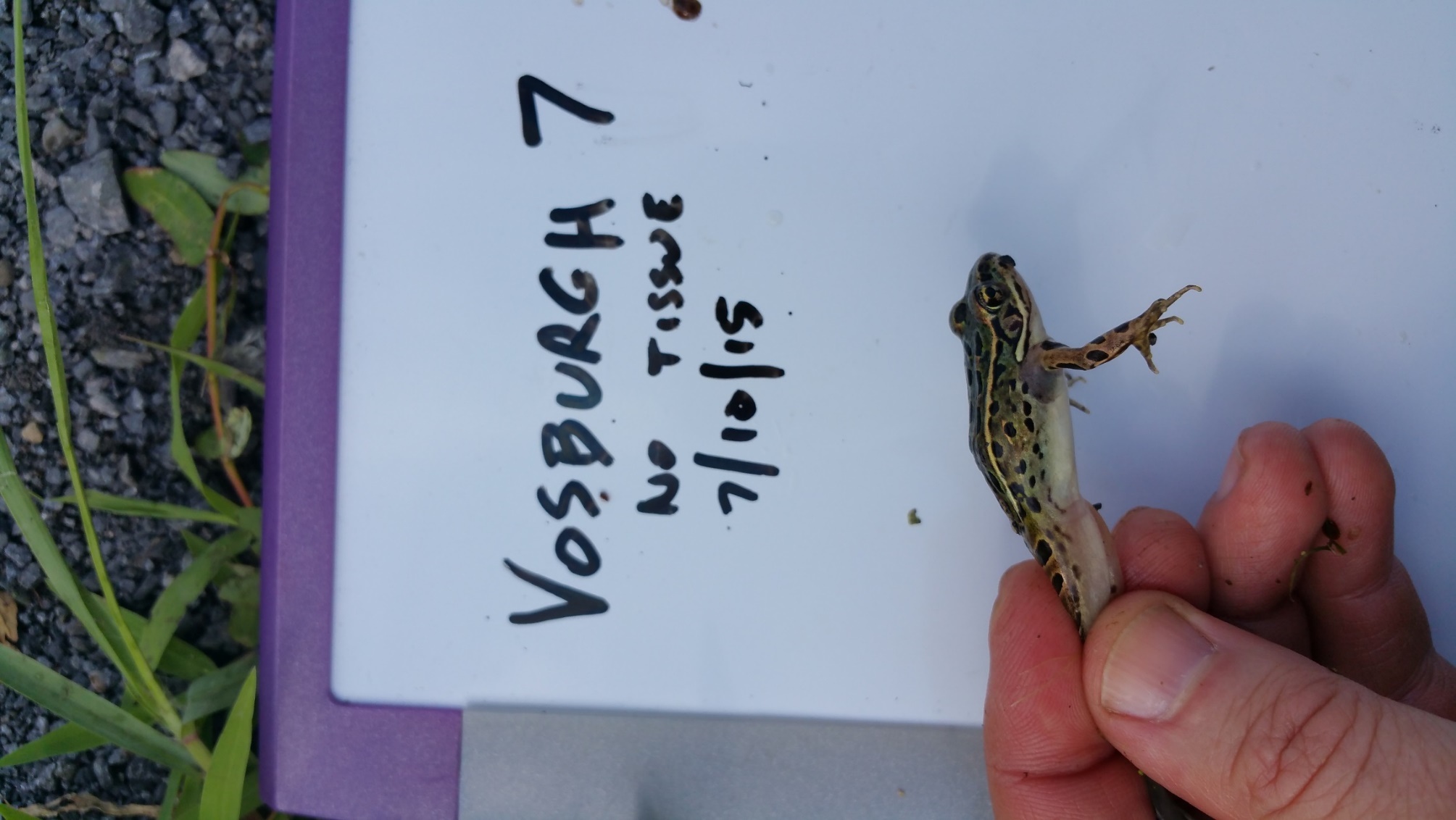 | 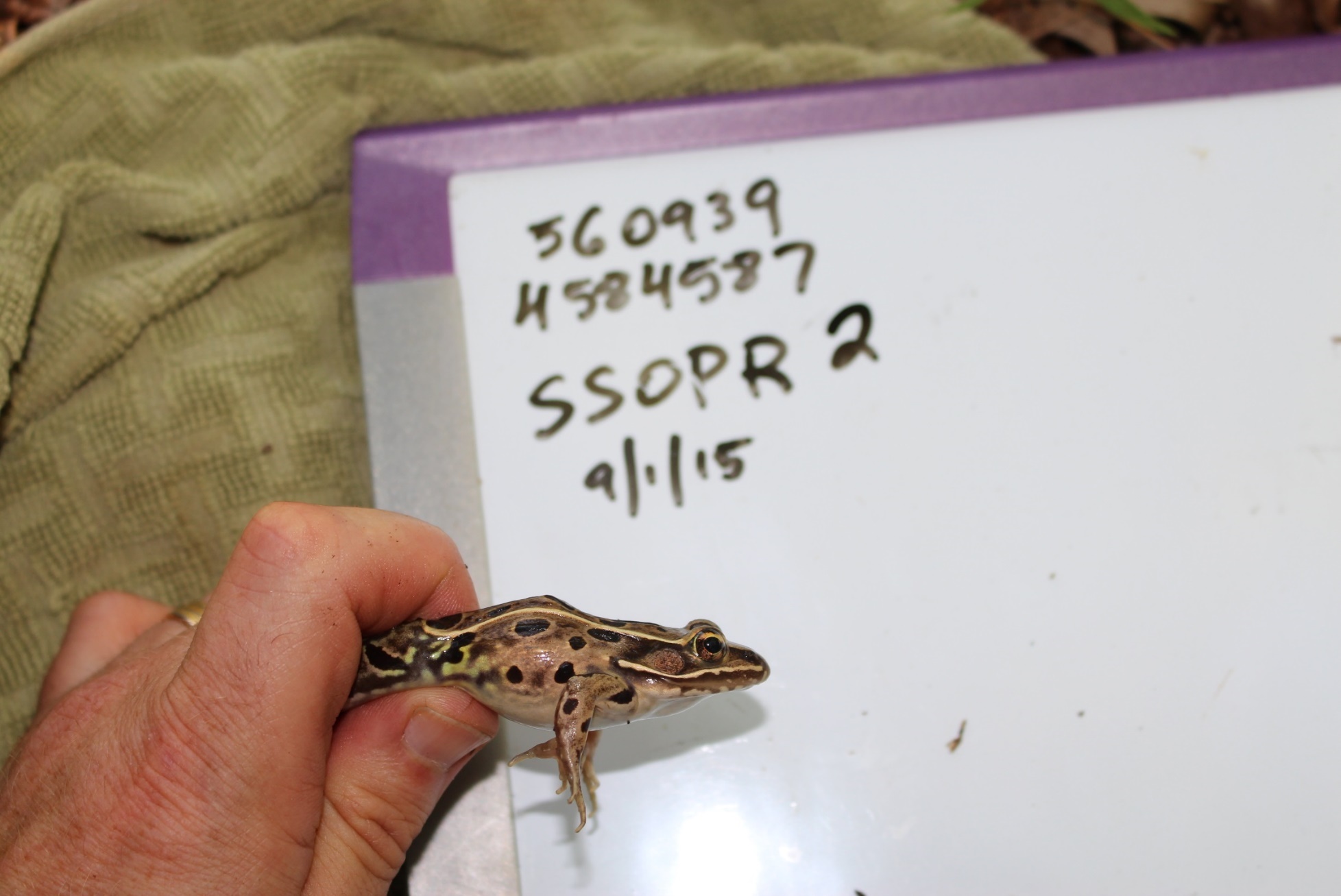 | 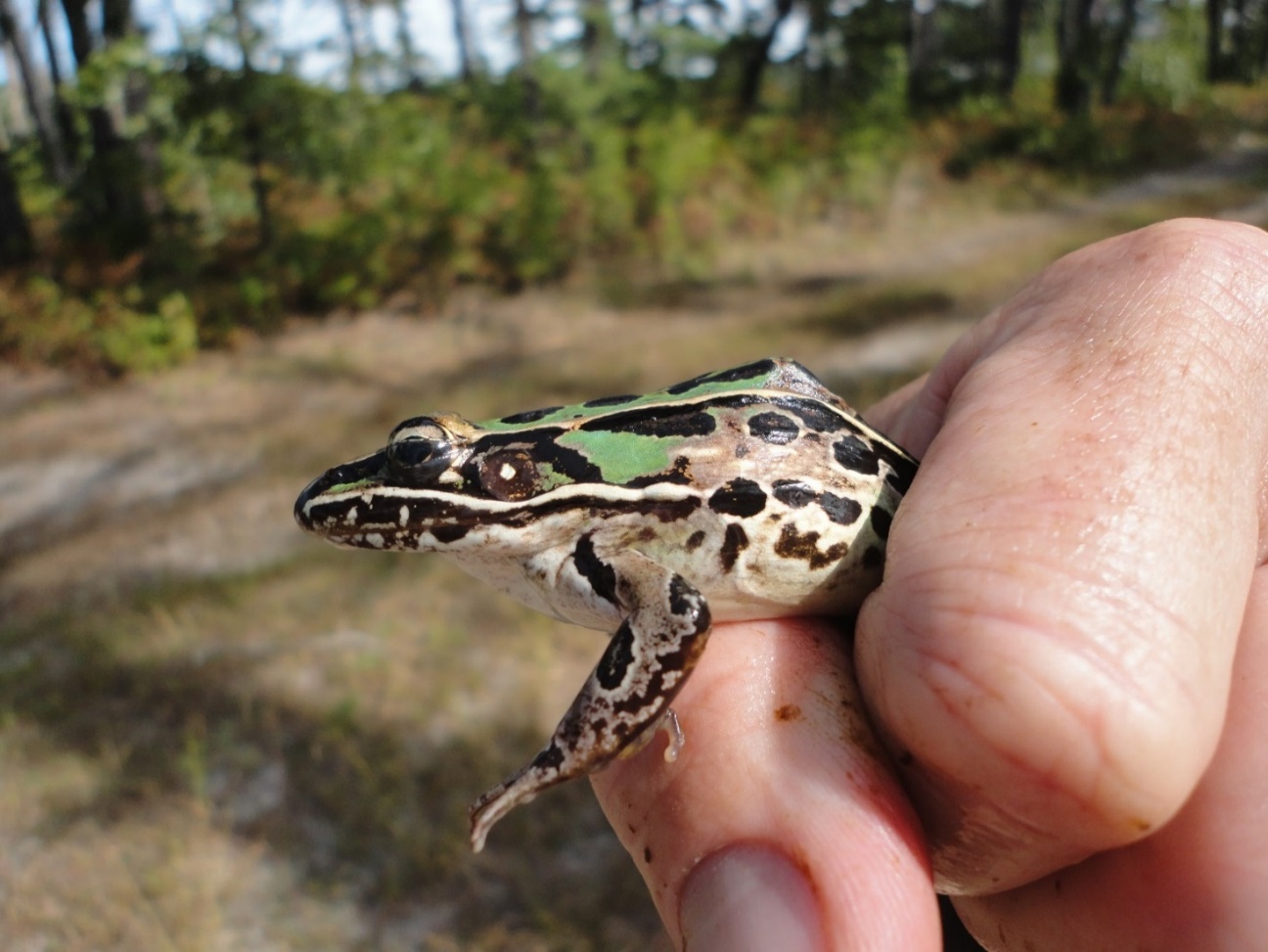 | 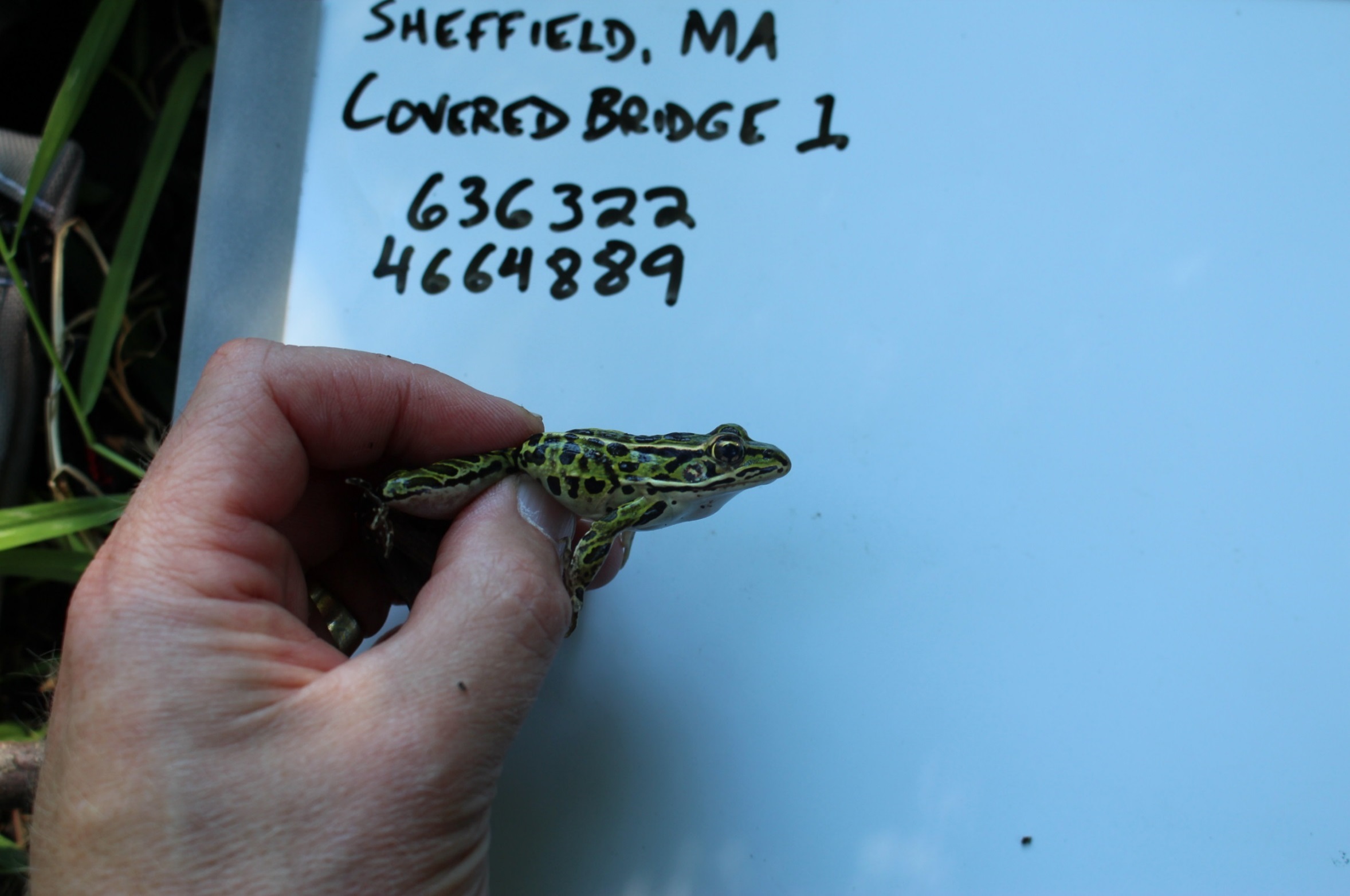 |
| None | None | None | Sharp dot, white/cream | Present; indistinct, green |

| **Femoral reticulum** | | |
| --- | --- | --- |
| 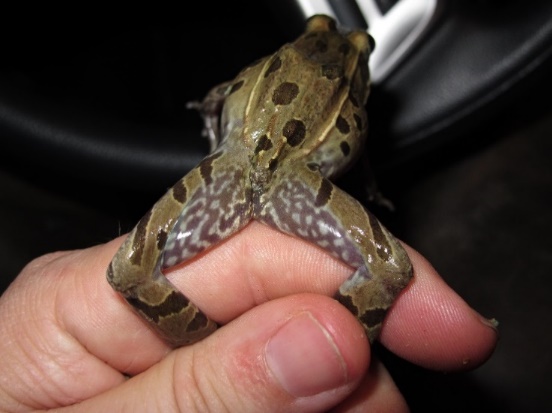 | 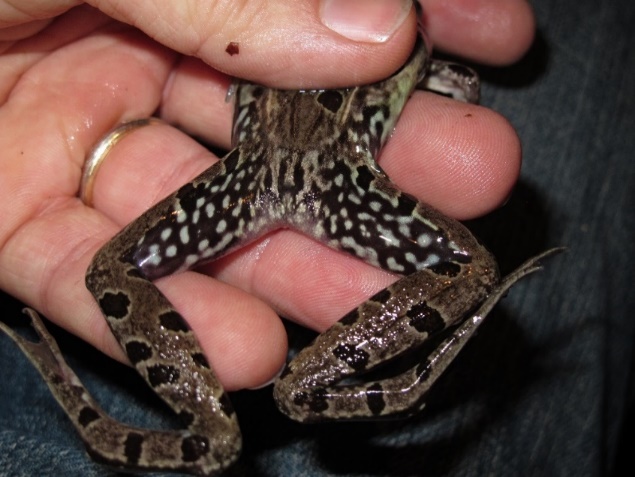 | 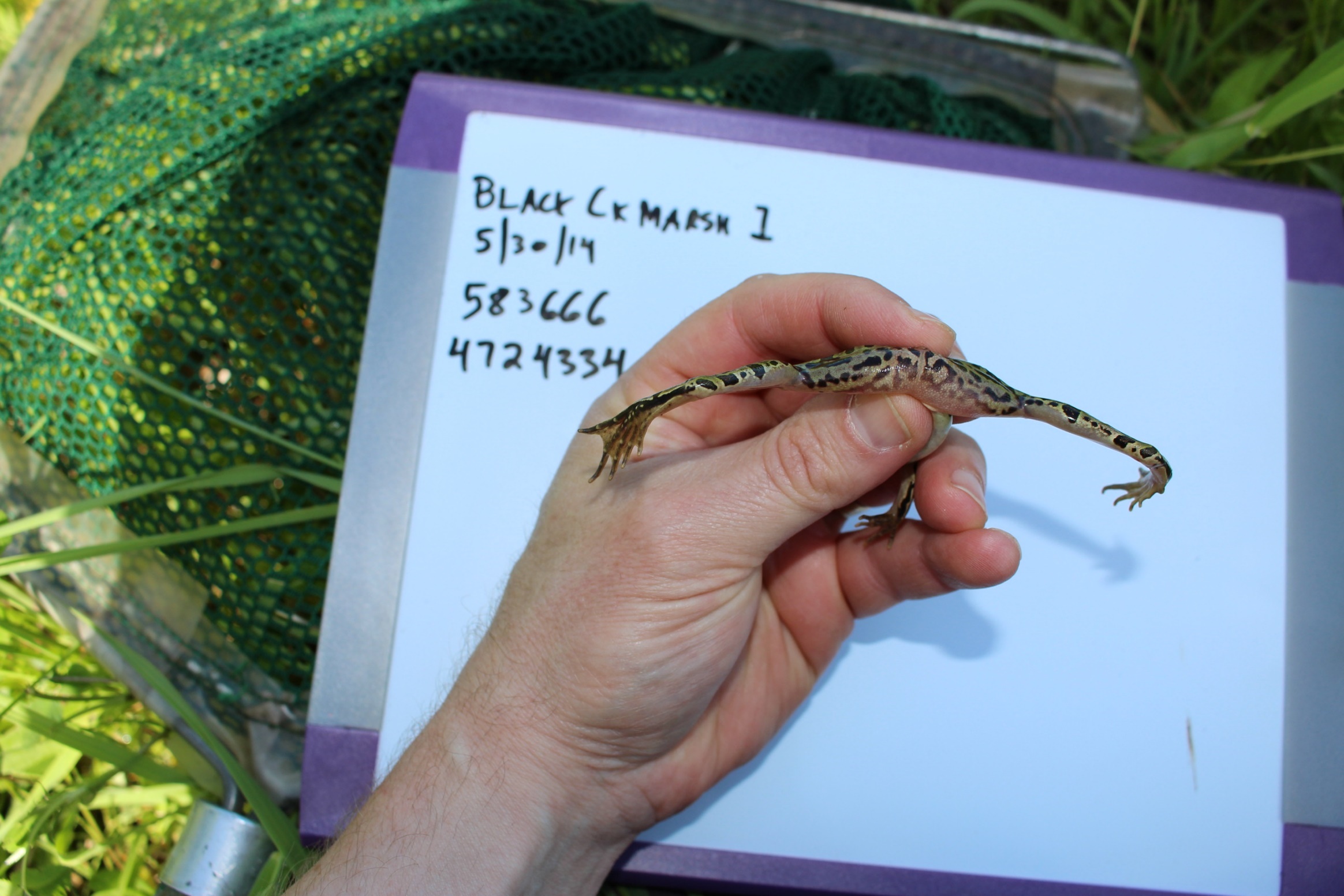 |
| Predominantly dark, many small blotches, cream | Predominantly dark, many small blotches, cream | Predominantly light, mostly large connected blotches, cream |
| 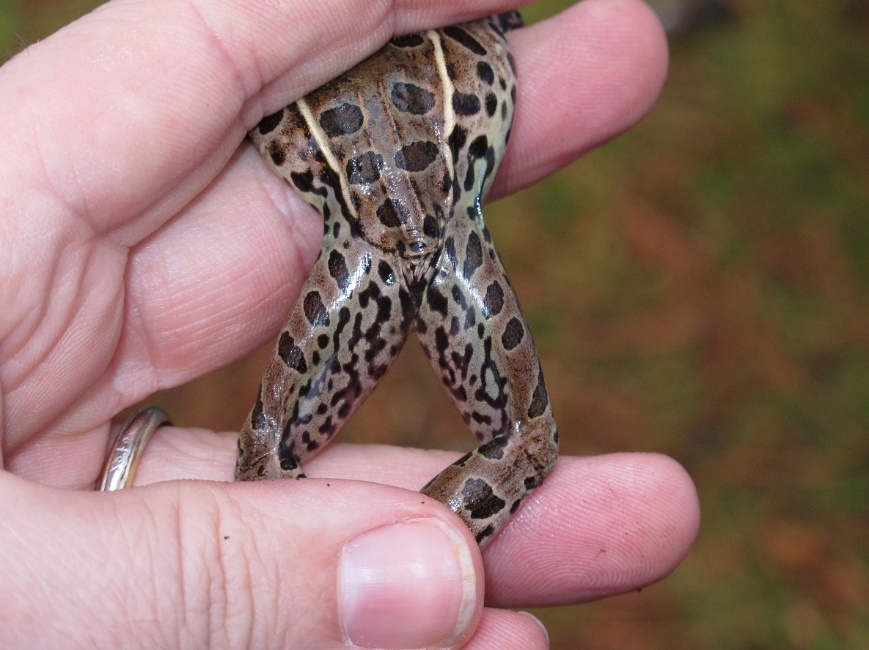 | 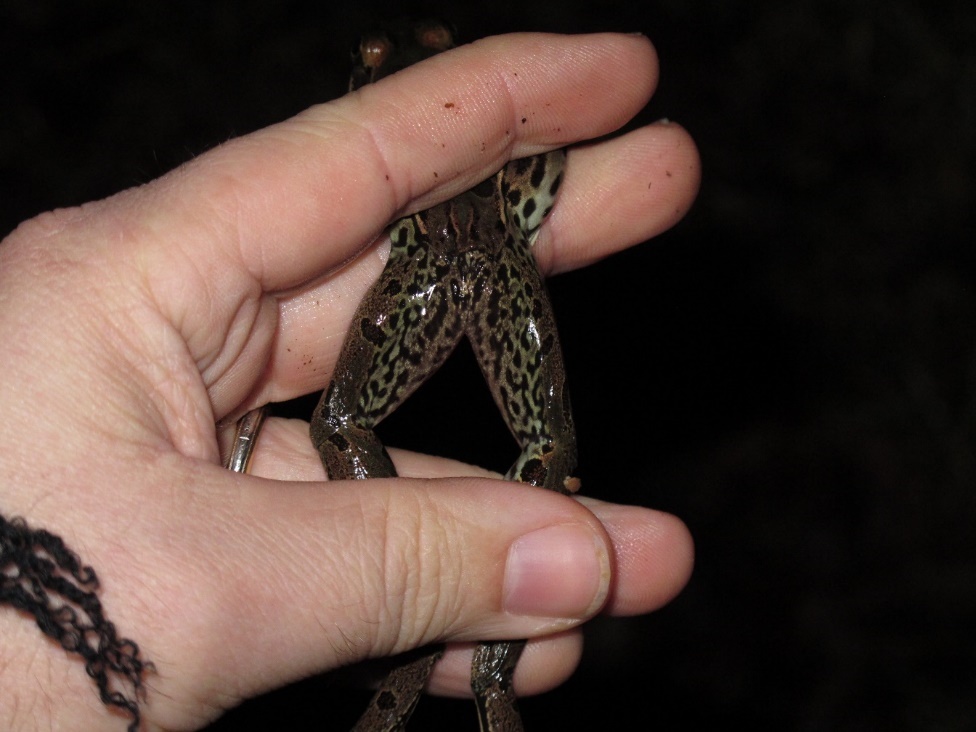 | 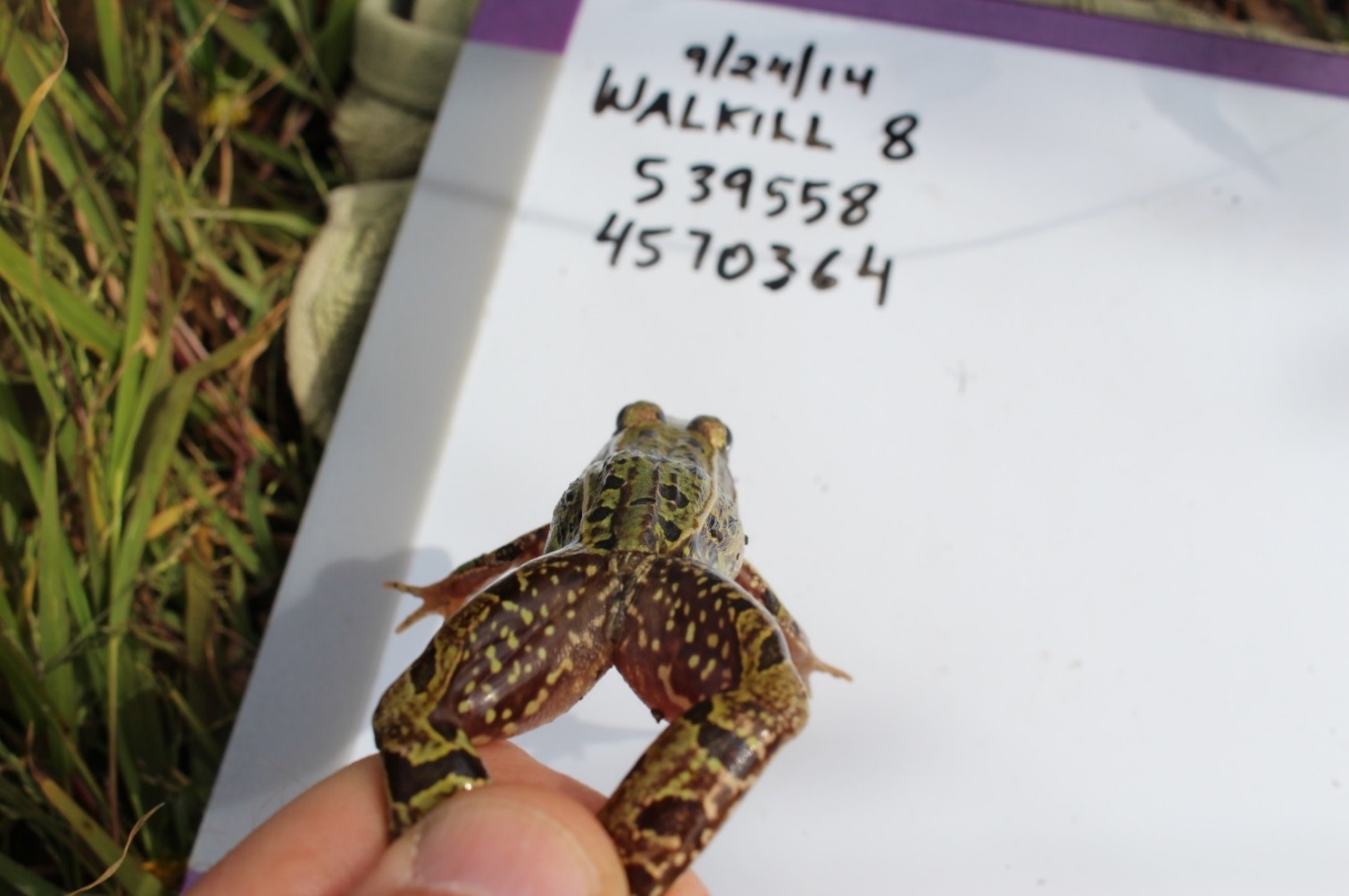 |
| Predominantly light, mostly large connected blotches, cream | Predominantly light, mostly large connected blotches, green | Predominantly dark, many small blotches, green |
| **Webbing of first toe on hind foot** | | |
| 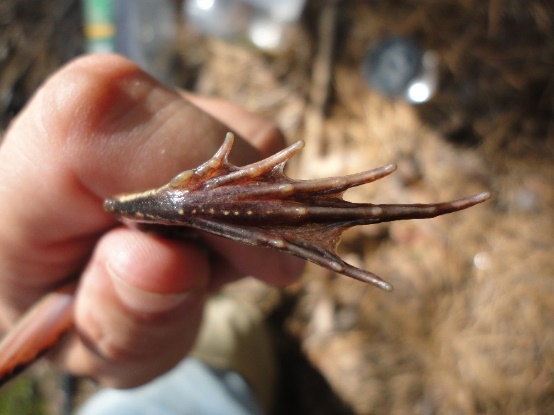 | 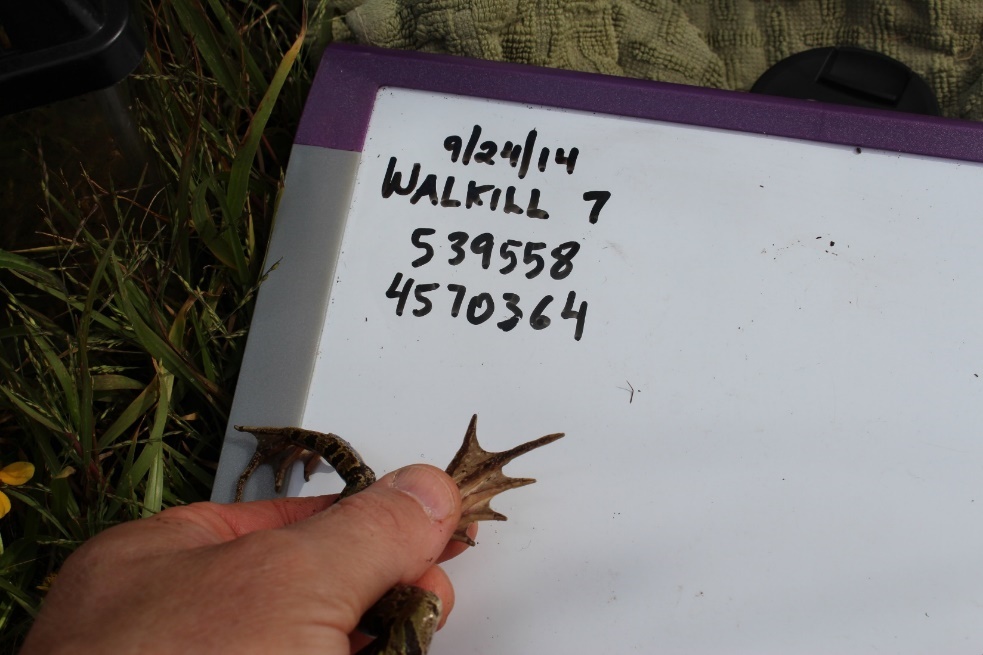 | 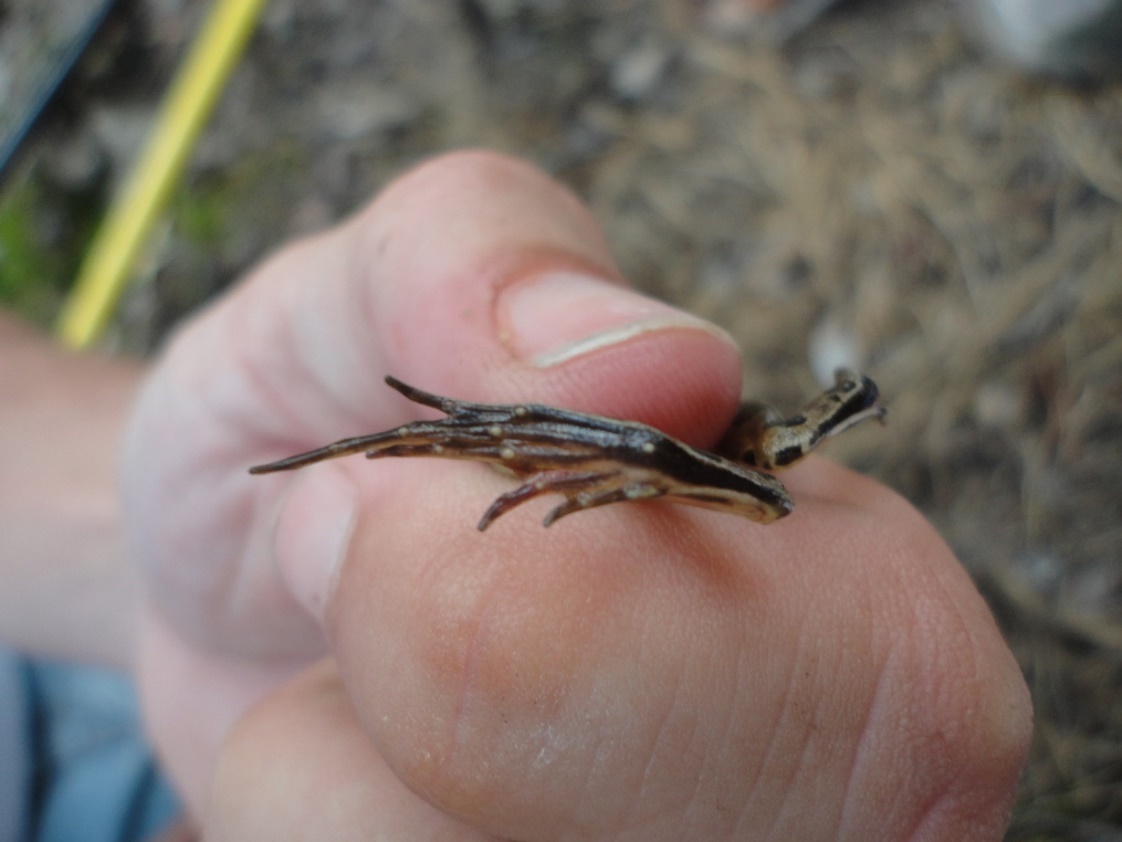 |
| Webbing on first toe (at bottom) stops midway up | Webbing on first toe (at top) curves to tip | Photo inconclusive |
