# Supplementary material for: Follow-up ecological studies for cryptic species discoveries: Decrypting the leopard frogs of the eastern U.S
Source: PLoS One. 2018 Nov 9;13(11):e0205805. doi: 10.1371/journal.pone.0205805 (PMC6226167; doi:10.1371/journal.pone.0205805)
Supplement: S2 Appendix — (DOCX) [file pone.0205805.s002.docx]

S2 Appendix. Reticulum characterization with ImageJ

For a more quantitative approach to characterizing femoral reticula coloration, we imported photographs with a clear view of the reticulum into ImageJ (108) and selected the dorsal thigh area with the greatest area exposed. We excluded photos with flash glare on the reticulum, ones that did not show approximately 75% of the reticulum, and ones depicting the reticulum as so dark that the software did not distinguish light and dark. We processed the selected area with the binary function to determine the percent of the reticulum that was dark.

We quantified the degree of darkness of femoral reticula of 55 frogs genetically identified as *R. kauffeldi* and 50 frogs genetically identified as *R. sphenocephala*. Reticula of *R. kauffeldi* were determined to be 70% dark on average, significantly darker than those of *R. sphenocephala*, which were around 55% dark on average (F_103,1_ = 52.38, *P* < 0.0001), although there was considerable overlap between species (S2 Fig). We also compared these values against our categorical ratings. While we underestimated the percent darkness overall in our ratings—both species on average were over 50% dark—our ratings generally matched up with the calculated percent dark. Reticula we called “predominantly dark” (*n* = 65) averaged 69.5% dark, while those we called “predominantly light” (*n* = 36) averaged 50.0% dark. There were a few frogs whose measured reticular darkness contradicted their categorical placement, but this comparison was imperfect because the categorical method may have relied on different or additional photographs than the ones used in the ImageJ analysis.


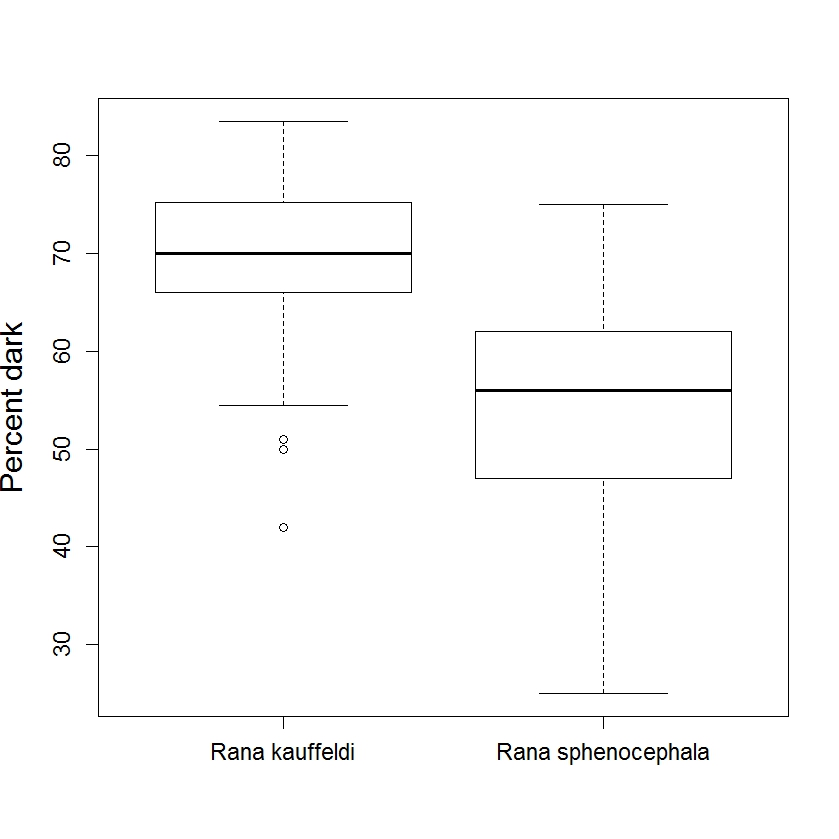


S2 Fig. Boxplot of percent of femoral reticulum classified as “dark” by ImageJ software for *Rana kauffeldi* (n = 55) and *R. sphenocephala* (n = 50) in the coastal northeastern U.S. Open circles are statistical outliers.
