# Supplementary material for: Follow-up ecological studies for cryptic species discoveries: Decrypting the leopard frogs of the eastern U.S
Source: PLoS One. 2018 Nov 9;13(11):e0205805. doi: 10.1371/journal.pone.0205805 (PMC6226167; doi:10.1371/journal.pone.0205805)
Supplement: S3 Appendix — (DOCX) [file pone.0205805.s003.docx]

S3 Appendix. Additional genetic methods and results.

To ensure results of the Structure analyses were not biased by including phased haplotypes with a posterior probability threshold of ≥0.80, which might be viewed as too low, we created two data sets, one with a minimum PHASE haplotype probability of 0.80 and one with a more conservative PHASE haplotype probability of 0.90. In each case, the remaining lower-probability haplotypes coded as null alleles. Phase input files were formatted [1], Phase version 2.1.1. [2,3] was run with default parameters, and Structure input files were formatted based on phased haplotypes with 13% or 15% null alleles for the Phase 80% and 90% data sets respectively. Following Newman et al. [4], we used Structure 2.3.4 [5] to determine the number of genetically distinct clusters (K) in for each data set. We used the admixture model [5] and assumed correlation of allele frequencies among clusters [6] and no other *a priori* population information. We ran 10 independent iterations of K values from 1 to 10. Iterations were run for 100,000 generations after a burn-in of 100,000 generations. For both datasets, the appropriate K value was chosen by using the Evanno method [7] visualized using Structure Harvester [8] which was also used to combine the results of multiple independent Structure iterations using CLUMPP [9].

To confirm genetic clustering from Structure analyses using a phased-haplotype independent approach, we generated a concatenated phylogeny for the five nuclear loci. Relationships among all sampled frogs were inferred using maximum likelihood (ML) inference in RAxML 8.2.10 and the GTRGAMMA model for sequence evolution [10] where each gene was independently partitioned by codon position (15 partitions in total). The phylogeny was rooted with a single chimeric (not all sequences were from the same individual) *R. catesbeiana* obtained from GenBank (CXCR4: AB612041.1; RAG-1: AB612037.1; SIA: DQ282778.1; TYR: AB612039.1). Branch support for each node was generated using the automatic bootstrap function with rapid bootstrapping on the CIPRES science gateway [10-12], which uses a stopping rule to calculate when sufficient replications have been completed. As most bootstrap values were very low, the analysis was re-run with 1000 bootstrap replicates with no difference in clade support.

The results of Structure analyses from both the 80% and 90% probability Phase data sets were quantitatively and qualitatively similar (S3 Fig1). The ΔK method of Evanno et al. [7] decisively found K=3 as the optimal number of clusters for both data sets (lnL = 4313.60, DK = 130.93; lnL =4138.27, DK = 141.15 for 80% and 90% respectively). Assigning species identification based on individuals included in these analyses from Newman et al. [4], resulted in qualitatively very similar results of 113 *R. kauffeldi*, 79 *R. sphenocephala*, 71 *R. pipiens*, and 36 “admixed” for 80% and 107 *R. kauffeldi*, 77 *R. sphenocephala*, 72 *R. pipiens*, and 34 “admixed” for 90% Phase haplotypes with the main differences seemingly resulting in differences of null haplotypes at the 90% probability threshold, and most changes being between “pure” and “pure-admixed” (i.e., “*R. kauffeldi*” to “admixed *R. kauffeldi*” or vice-versa). Overall, these results suggest that 80% and 90% thresholds return very similar results, and we chose to use 80% since it allowed us to categorize a few more individuals.

The concatenated maximum likelihood phylogeny identified three major, but poorly supported, clades representing *R. kauffeldi, R. pipiens,* and *R. sphenocephala* (S3 Fig 2). A handful of individuals failed to be included in one of these three clades, and were positioned branching from internal stems in the phylogeny. These were individuals shown to be potentially “admixed” in the Structure analyses or ones that had large amounts of missing data and likely weaker support for topological positioning in the phylogeny.


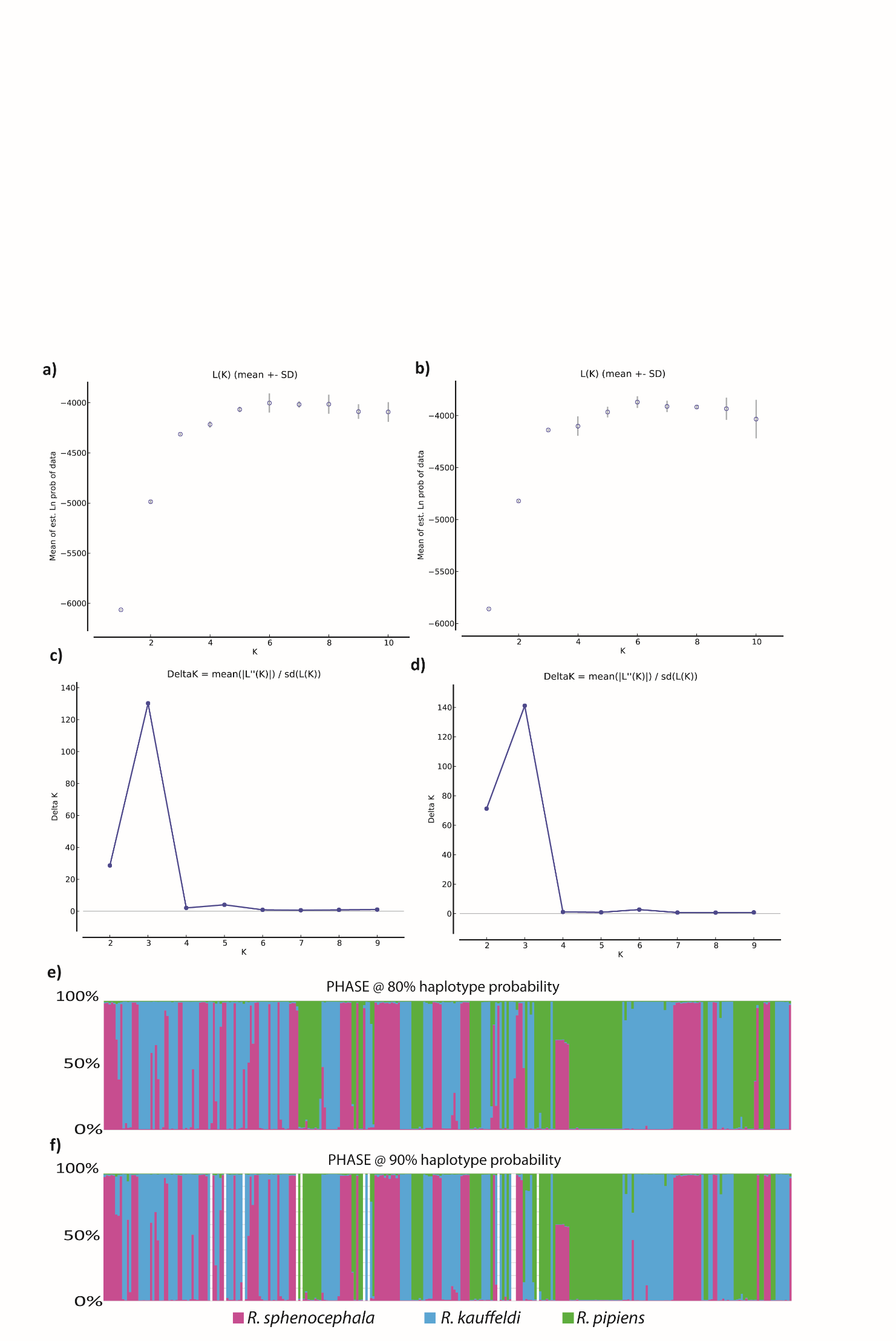


S3 Fig 1. Plots showing the mean ln probability of K given the data (+/- standard deviation) for Structure output from Phased haplotypes at minimum probability of 80% (a) or 90% (b) and delta ln probability for increasing values of K from Phased haplotypes with a minimum probability of 80% (c) or 90% (d). Graphs modified from results generated in (8). Results of Structure population assignments for Phased haplotypes at minimum probability of 80% (e) or 90% (f) with individuals ordered alphanumerically showing qualitative similarity between analyses. Individuals lost due to high null alleles at a Phase probability of 90% are shown by white bars. Individuals are in the same order in both plots.


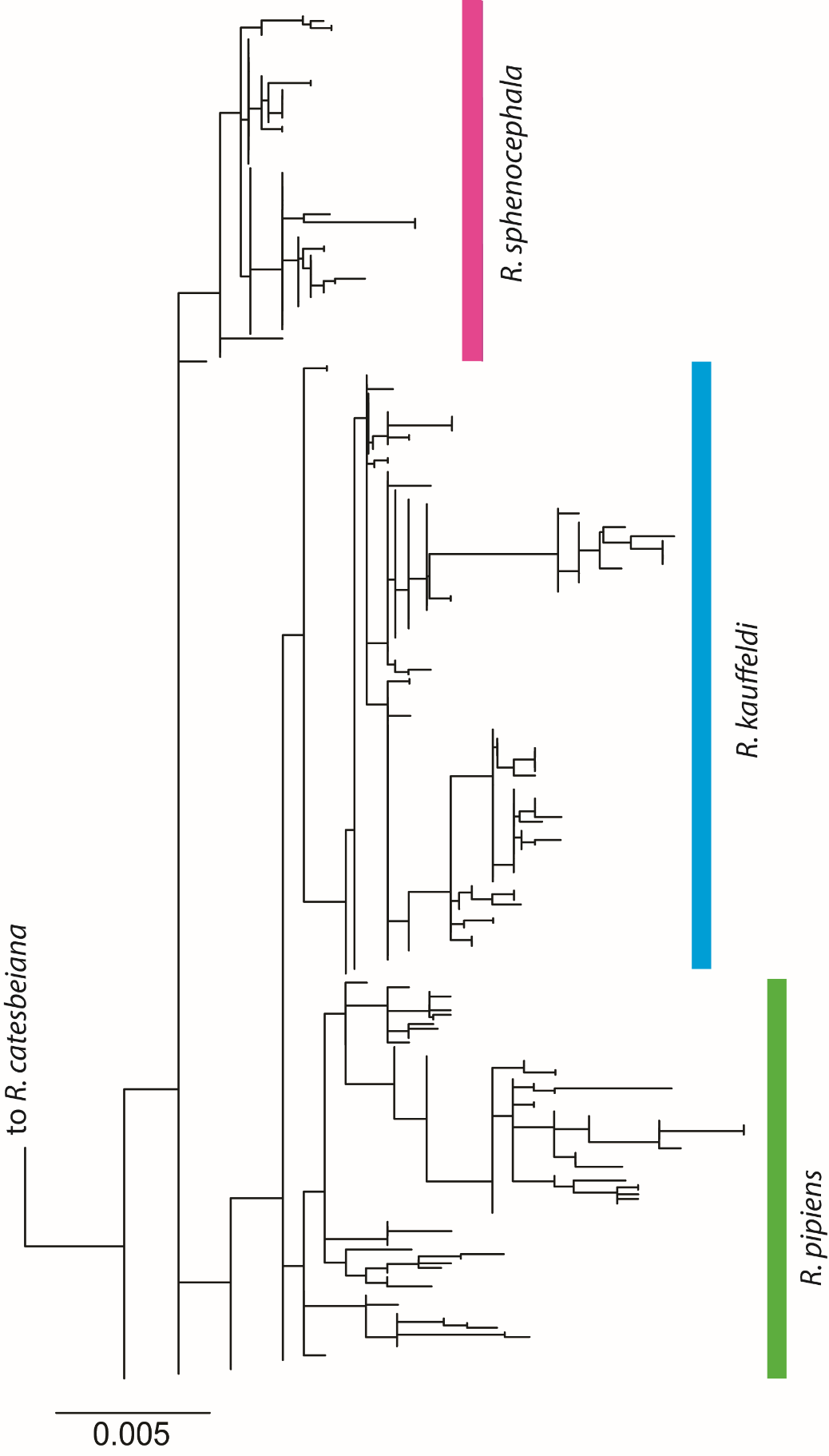


S3 Fig 2. A concatenated maximum likelihood phylogeny showing the major clade assignments and relationships of frogs included in this study. All major nodes received bootstrap scores <50 and individual IDs are removed for clarity.

**Supplemental Citations**

1. Flot JF. SeqPHASE: a web tool for interconverting PHASE input/output files and FASTA sequence alignments. Molecular Ecology Resources. 2010 Jan;10(1):162-6.
2. Stephens M, Smith NJ, Donnelly P. A new statistical method for haplotype reconstruction from population data. The American Journal of Human Genetics. 2001 Apr;68(4):978–89.
3. Stephens M, Donnelly P. A comparison of Bayesian methods for haplotype reconstruction from population genotype data. The American Journal of Human Genetics. 2003 Nov;73(5):1162–9.
4. Newman CE, Feinberg JA, Rissler LJ, Burger J, Shaffer HB. A new species of leopard

frog (Anura: Ranidae) from the urban northeastern US. Molecular Phylogenetics and

Evolution. 2012 May;63(2):445–55.

1. Pritchard JK, Stephens M, Donnelly P. Inference of population structure using multilocus

genotype data. Genetics. 2000 Jun 1;155(2):945–59.

1. Falush D, Stephens M, Pritchard JK. Inference of population structure using multilocus genotype data: linked loci and correlated allele frequencies. Genetics. 2003 Aug 1;164(4):1567-87.
2. Evanno G, Regnaut S, Goudet J. Detecting the number of clusters of individuals using the software STRUCTURE: a simulation study. Molecular ecology. 2005 Jul 1;14(8):2611-20.
3. Earl DA, vonHoldt BM. STRUCTURE HARVESTER: a website and program for

visualizing STRUCTURE output and implementing the Evanno method. Conservation Genet Resour. 2012 Jun 1;4(2):359–61.

1. Jakobsson M, Rosenberg NA. CLUMPP: a cluster matching and permutation program for dealing with label switching and multimodality in analysis of population structure. Bioinformatics. 2007 May 7;23(14):1801-6.
2. Miller MA, Pfeiffer W, Schwartz T. Creating the CIPRES Science Gateway for inference of large phylogenetic trees. InGateway Computing Environments Workshop (GCE), 2010 2010 Nov 14 (pp. 1-8). Ieee.
3. Pattengale ND, Alipour M, Bininda-Emonds OR, Moret BM, Stamatakis A. How many bootstrap replicates are necessary?. Journal of computational biology. 2010 Mar 1;17(3):337-54.
4. Stamatakis A. RAxML version 8: a tool for phylogenetic analysis and post-analysis of large phylogenies. Bioinformatics. 2014 May 1;30(9):1312-3.
