# Supplementary material for: Follow-up ecological studies for cryptic species discoveries: Decrypting the leopard frogs of the eastern U.S
Source: PLoS One. 2018 Nov 9;13(11):e0205805. doi: 10.1371/journal.pone.0205805 (PMC6226167; doi:10.1371/journal.pone.0205805)
Supplement: S4 Appendix — Frogs were identified using genetics and designated as “pure” if they had a 90% or greater match with a single species; individuals with a species match between 10% and 90% were designated as admixed. Sample sizes for each character are given because not every frog had photographs suitable for examination of that character. (DOCX) [file pone.0205805.s004.docx]

S4 Appendix. Morphological and color characters from photographic analysis of 220 leopard frogs (*Rana* spp.) in the eastern U.S. Frogs were identified using genetics and designated as “pure” if they had a 90% or greater match with a single species; individuals with a species match between 10% and 90% were designated as admixed. Sample sizes for each character are given because not every frog had photographs suitable for examination of that character.

|  |  | | Species | | | | | |
| --- | --- | --- | --- | --- | --- | --- | --- | --- |
| Character | Character state | Pure *R. kauffeldi*  (n = 80) | | Admixed *R. kauffeldi*  (n = 16) | Pure *R.* *pipiens*  (n = 45) | Admixed  *R. pipiens*  (n = 5) | Pure *R. sphenocephala*  (n = 64) | Admixed *R. sphenocephala*  (n = 10) |
| Dorsal spots | Min | 7 | | 6 | 11 | 12 | 6 | 8 |
|  | Max | 25 | | 29 | 28 | 23 | 36 | 24 |
|  | Mean ± sd | 13.6 ± 3.7 | | 15.5 ± 6.3 | 18.1 ± 4.2 | 16.7 ± 5.7 | 17.0 ± 5.7 | 14.0 ± 4.7 |
|  |  |  | |  |  |  |  |  |
|  | Mainly larger than eye | 12 | | 5 | 27 | 3 | 19 | 1 |
|  | Mainly smaller than eye | 66 | | 11 | 15 | 0 | 43 | 9 |
|  |  |  | |  |  |  |  |  |
| Snout spot | Absent | 67 | | 12 | 12 | 2 | 54 | 9 |
|  | Small | 6 | | 2 | 0 | 0 | 8 | 0 |
|  | Large | 6 | | 1 | 30 | 2 | 1 | 1 |
|  |  |  | |  |  |  |  |  |
| Snout | Blunt | 28 | | 4 | 16 | 2 | 6 | 1 |
|  | Intermediate | 3 | | 2 | 7 | 1 | 7 | 0 |
|  | Pointed | 14 | | 5 | 16 | 1 | 32 | 2 |
|  |  |  | |  |  |  |  |  |
| Reticulum | Predominantly dark | 75 | | 12 | 0 | 0 | 14 | 5 |
|  | About 50/50 | 1 | | 0 | 42 | 5 | 6 | 3 |
|  | Predominantly light | 1 | | 1 | 0 | 0 | 44 | 1 |
|  |  |  | |  |  |  |  |  |
|  | Mostly large, connected blotches | 12 | | 3 | 34 | 4 | 55 | 5 |
|  | Mostly small, unconnected dots | 64 | | 8 | 8 | 1 | 9 | 4 |
|  |  |  | |  |  |  |  |  |
| Tympanum spots | None | 2 | | 1 | 2 | 0 | 0 | 0 |
|  | 1 sharp, 1 indistinct | 4 | | 1 | 4 | 1 | 3 | 2 |
|  | 2 indistinct | 9 | | 1 | 5 | 0 | 2 | 0 |
|  | 2 sharp | 21 | | 7 | 17 | 0 | 53 | 6 |
|  | Left spot absent | 8 | | 2 | 1 | 0 | 4 | 0 |
|  | Left spot indistinct | 18 | | 3 | 6 | 1 | 3 | 2 |
|  | Left sharp blotch | 12 | | 5 | 21 | 1 | 30 | 3 |
|  | Left sharp dot | 18 | | 4 | 4 | 0 | 26 | 5 |
|  |  |  | |  |  |  |  |  |
|  | Right spot absent | 8 | | 2 | 4 | 2 | 0 | 0 |
|  | Right spot indistinct | 15 | | 1 | 16 | 0 | 4 | 1 |
|  | Right sharp blotch | 12 | | 5 | 17 | 2 | 27 | 2 |
|  | Right sharp dot | 25 | | 5 | 4 | 0 | 32 | 5 |
|  |  |  | |  |  |  |  |  |
|  | Left spot brown/bronze | 12 | | 3 | 15 | 1 | 3 | 3 |
|  | Left spot green | 11 | | 4 | 6 | 0 | 11 | 2 |
|  | Left spot white/cream | 22 | | 5 | 9 | 1 | 44 | 5 |
|  |  |  | |  |  |  |  |  |
|  | Right spot brown/bronze | 14 | | 3 | 19 | 1 | 2 | 3 |
|  | Right spot green | 13 | | 6 | 10 | 0 | 11 | 2 |
|  | Right spot white/cream | 23 | | 2 | 5 | 1 | 48 | 4 |
|  |  |  | |  |  |  |  |  |
| Combinations | Dark reticulum, large light blotches | 10 | | 2 | 0 | 0 | 8 | 1 |
|  | Dark reticulum, small light dots | 64 | | 8 | 0 | 0 | 6 | 4 |
|  | Dark retic, 1 tymp spot sharp, 1 blurry | 3 | | 0 | 0 | 0 | 0 | 2 |
|  | Dark retic, 2 tymp spots sharp | 21 | | 5 | 0 | 0 | 12 | 2 |
|  | Dark retic, 2 tymp spots blurry | 9 | | 1 | 0 | 0 | 0 | 0 |
